# Genome‐Wide Aggregated *Trans* Effects Analysis Identifies Genes Encoding Immune Checkpoints as Core Genes for Rheumatoid Arthritis

**DOI:** 10.1002/art.43125

**Published:** 2025-03-16

**Authors:** Athina Spiliopoulou, Andrii Iakovliev, Darren Plant, Megan Sutcliffe, Seema Sharma, Cankut Cubuk, Myles Lewis, Costantino Pitzalis, Anne Barton, Paul M. McKeigue

**Affiliations:** ^1^ University of Edinburgh Edinburgh Scotland; ^2^ University of Manchester and the National Institute for Health and Care Research Manchester Biomedical Research Centre, Manchester University NHS Foundation Trust Oxford Road Manchester United Kingdom; ^3^ University of Manchester Manchester United Kingdom; ^4^ Queen Mary University of London and Barts Health NHS Trust and NIHR Barts Biomedical Research Centre London United Kingdom

## Abstract

**Objective:**

The sparse effector “omnigenic” hypothesis postulates that the polygenic effects of common single nucleotide polymorphisms (SNPs) on a typical complex trait are mediated by *trans* effects that coalesce on expression of a relatively sparse set of core genes. The objective of this study was to identify core genes for rheumatoid arthritis by testing for association of rheumatoid arthritis with genome‐wide aggregated *trans* effects (GATE) scores for expression of each gene as transcript in whole blood or as circulating protein levels.

**Methods:**

GATE scores were calculated for 5,400 cases and 453,705 non‐cases of primary rheumatoid arthritis in UK Biobank participants of European ancestry.

**Results:**

Testing for association with GATE scores identified 16 putative core genes for rheumatoid arthritis outside the HLA region, of which six—*TP53BP1*, *PDCD1*, *TNFRSF14*, *LAIR1*, *LILRA4*, and *IDO1*—were supported by Mendelian randomization analysis based on the marginal likelihood of the causal effect parameter. Five of these 16 genes were validated by a reported association of rheumatoid arthritis with SNPs within 200 kb of the transcription site, eight by association of the measured protein level with rheumatoid arthritis in UK Biobank, 10 by experimental perturbation in mouse models of inflammatory arthritis, and two—*CTLA4* and *PDCD1*—by evidence that drugs targeting the gene cause or ameliorate inflammatory arthritis in humans. Fourteen of these 16 genes are in pathways affecting immunity or inflammation, and six—*CD5*, *CTLA4*, *TIGIT*, *LAIR1*, *TNFRSF14*, and *PDCD1*—encode receptors that have been characterized as immune checkpoints exploited by cancer cells to escape the immune response.

**Conclusion:**

These results highlight the key role of immune checkpoints in rheumatoid arthritis and identify possible therapeutic targets.

## INTRODUCTION

Rheumatoid arthritis is an immune‐mediated inflammatory disease with a prevalence of about 1% in most populations. The genetic information for discrimination, equal to the logarithm to base two of the recurrence risk ratio in first‐degree relatives,^1^ is about 2.3 bits,^2^ of which the HLA region accounts for about 0.8 bits.^3^ Although the catalog of genome‐wide association studies (GWAS) lists 337 genomic regions outside the HLA region that contain single nucleotide polymorphisms (SNPs) associated with rheumatoid arthritis at the conventional genome‐wide threshold of *P* < 5 × 10^−8^, the genes in these regions are mostly broadly expressed and are not in pathways specifically relevant to immune‐mediated disease.^4^ Although the treatment of rheumatoid arthritis has been revolutionized by drugs that target specific proteins mediating inflammation, the contribution of GWAS to discovery of drug targets for rheumatoid arthritis has been limited.^5^

Conventional GWAS analyses focus on identifying the nearby genes (usually within 200 kb) that mediate the effects of common variants on disease through *cis* effects. However, for a typical gene, most of the SNP heritability of levels of the transcript or encoded protein is attributable to *trans* effects of variants that are distant from the transcription site.^6^ On this basis, the “omnigenic” sparse effector model was proposed as a fundamental rethink of the genetic architecture of complex traits.^7^ This postulates that most of the polygenic effects on a typical complex trait are mediated through weak *trans* effects of common variants that coalesce on expression of a relatively sparse set of “core” effector genes in relevant tissues. The availability of summary statistics from large GWAS studies of transcripts in whole blood or proteins in plasma has made it possible to test this hypothesis by constructing genotypic predictors of gene expression based on aggregated *trans* effects and testing these genotypic scores for association with the disease or trait under study. We have reported an application of this genome‐wide aggregated *trans* effects (GATE) analysis pipeline to type 1 diabetes, which identified a set of putative core genes regulating the differentiation and activity of CD4+ Treg cells.^8^ The objective of this study was to investigate whether GATE analysis can identify core genes for rheumatoid arthritis.

## MATERIALS AND METHODS

### Ethical approval

Ethical approval for the UK Biobank was previously obtained from the North West Centre for Research Ethics Committee (11/NW/0382). Informed consent was obtained for all study participants. The work described herein was approved by the UK Biobank under application 23652. Ethical approval for the transcriptomics study was granted by the North West 6 Central Manchester South Research Ethics Committee (COREC 04/Q1403/37). All patients provided written consent.

### Case definition

The case definition of rheumatoid arthritis in the UK Biobank cohort was based on any of three criteria: (1) hospital discharge or death certificate with an International Classification of Diseases diagnostic code for rheumatoid arthritis; (2) any primary care diagnosis of rheumatoid arthritis; or (3) self‐reported diagnosis of rheumatoid arthritis supported by prescription of a disease‐modifying antirheumatic drug at baseline or during follow‐up. Of 487,152 individuals with nonmissing phenotype and genotype data, 5,958 had ever been diagnosed with rheumatoid arthritis. When those with a diagnosis of Sjögren's disease antedating the diagnosis of rheumatoid arthritis were excluded, 5,731 cases of primary rheumatoid arthritis remained. The full dataset was pruned to ensure that no pairs of individuals with kinship coefficient >0.05 remained. As summary statistics for *cis* and *trans* effects of SNP genotypes on gene expression were available only from studies of individuals of European ancestry, the dataset for *trans* effects analysis was restricted to those participants who reported their ethnic origin as white. After applying these exclusions, there were 451,447 individuals, of whom 5,292 were classified as cases. Of these, 2,015 were classified as incident cases, on the basis that the first mention of a diagnosis of rheumatoid arthritis was later than the date on which they were assessed.

### GATE analysis

**Figure 1:**
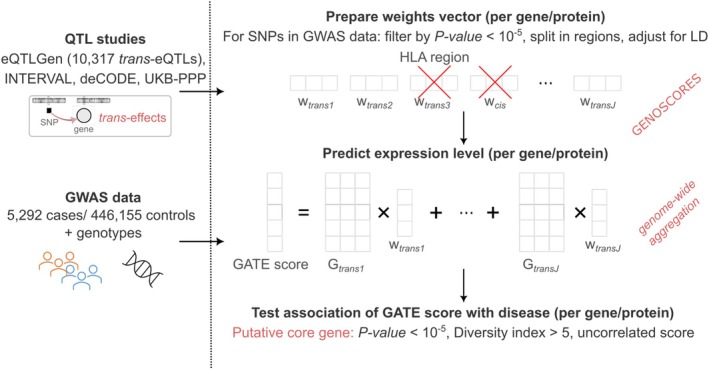
Graphic illustration of the data sources and analytical steps for identification of core genes for rheumatoid arthritis. eQTL, expression QTL; GATE, genome‐wide aggregated *trans* effects; GWAS, genome‐wide association study; LD, linkage disequilibrium; QTL, quantitative trait locus; SNP, single nucleotide polymorphism; UKB‐PPP, UK Biobank Pharma Proteomics Project.

Methods for GATE analysis have been described previously.^8^ The data sources and analytical steps are illustrated in Figure 1. The list of SNPs that were typed or imputed in UK Biobank was uploaded to the GENOSCORES server. A database query extracts the univariate coefficients of regression of expression of each target gene on each of these SNPs, filtered by setting a threshold of *P* < 10^−5^. For each target gene and each clump of associated SNPs containing at least one SNP associated with expression of the gene at *P* < 10^−6^, the vector of coefficients is multiplied by the inverse correlation matrix among SNP genotypes computed from the 1000 Genomes reference panel to obtain a vector of multivariable weights that are corrected for linkage disequilibrium (LD). A pseudoinverse solution is implemented to handle ill‐conditioned matrices in clumps with highly correlated SNPs. For each gene and each clump of associated SNPs, a locus‐specific score is computed by multiplying the vector of adjusted weights by the matrix of SNP genotypes in the target dataset. The locus‐specific scores for each gene are summed over *trans*‐quantitative trait loci (QTLs) to obtain genome‐wide aggregated *trans* scores.

*Trans*‐expression QTL (*trans*‐eQTL) scores were computed from eQTLGen phase 1, in which only 10,317 trait‐associated SNPs were tested for *trans* associations.^9^ For these scores, the threshold for defining clumps of associated SNPs was relaxed to *P* < 10^−5^ because for these trait‐associated SNPs, the prior probability of an effect on gene expression is higher than it is for random SNPs. *Trans*‐protein QTL (*trans*‐pQTL) scores were computed from three studies of circulating proteins:

- 1,478 proteins on the SomaLogic panel measured in plasma on 3,301 blood donors in the INTERVAL study.^10^
- 4,719 proteins on the SomaLogic version 4 panel measured in plasma on 35,559 Icelanders in the deCODE study^11^; 2,207 aptamers on this platform that appeared to cross‐react with *CFH*, encoding complement factor H, were excluded. The criteria for identifying these aptamers were a *trans*‐pQTL at the *CFH* locus, no *cis*‐pQTL, and association of the *trans* score with age‐related macular degeneration.
- 2,923 proteins on the Olink Explore panel measured in plasma on 54,306 participants in UK Biobank.^12^

As the HLA region is a hotspot for *trans*‐QTLs for genes involved in immunity and inflammation^13^ and associations of these *trans*‐QTLs with autoimmune disease are heavily confounded by the direct effects of HLA antigens, the HLA region (from 25 to 34 Mb on chromosome 6) was excluded from the computation of genome‐wide *trans* scores. *Cis*‐eQTLs and *cis*‐pQTLs were excluded from the aggregated *trans* scores and tested for association with the disease separately.

### Statistical analysis

When testing for association with genotypic scores for Olink proteins calculated from the UK Biobank proteomics study, the 54,306 participants who were included in the proteomics study were excluded from tests of association of the genotypic scores with the outcome. A logistic regression model was fitted with rheumatoid arthritis as response variable with sex and the first 20 genotypic principal components as covariates. The fitted values from this null model were used to compute tests for association with the *cis* score and aggregated *trans* score predicting the levels of a transcript or circulating protein. These tests were computed as efficient score tests based on the gradient and second derivative of the log‐likelihood at the null. Log odds ratios in the tables have been standardized by multiplying them by the SD of the score so that the coefficient is the log odds ratio associated with an increase of the score by one SD.

As an index of the effective number of unlinked *trans*‐QTLs contributing to each genome‐wide *trans* score, we calculated the diversity index or Hill number.^14^ For each gene, the diversity index was computed from the variances *σ*_1_,…,*σ*_K_ of the *K* locus–specific *trans* scores as 2^−∑^_i_^*p*^_i_^log^_2_^*p*^_i_, where *p*_*i*_ = *σ*_*i*_^2^ / ∑_j_ *σ*_*j*_^2^. This index can take values from 1, if one of the QTLs has much larger variance than the others, to *K*, if the variances of the locus‐specific *trans* scores are equal.

For each putative core gene identified through aggregating the effects of at least 10 *trans*‐QTLs, an instrumental variable (“Mendelian randomization”) analysis was undertaken. This was based on constructing a scalar genetic instrument from each *trans*‐QTL and marginalizing over the distribution of direct (pleiotropic) effects of the instrument on the outcome to compute the likelihood of the causal effect parameter as described elsewhere.^15^ *Cis*‐QTLs were excluded from these analyses because *cis*‐acting SNPs frequently alter the splicing of the gene product so that effects on the measured level of transcript or circulating protein do not correspond to effects on function. A null result in these Mendelian randomization analyses does not exclude a causal effect because even with 10 or more instruments (*trans*‐QTLs), there may not be enough information in the data to learn the posterior distribution of direct effects.

### Filtering and validation criteria

As before,^8^ the GATE scores were filtered to retain only those scores for which the effective number of *trans*‐QTLs was greater than five. This retained 413 aggregated *trans*‐eQTL scores for 413 unique genes and 3,256 aggregated *trans*‐pQTL scores for 2,586 unique genes. For the initial list of putative core genes, we set a threshold of *P* < 10^−5^ for association of disease with GATE scores.

We defined six criteria for validation as a core gene:

1. Any SNP association with rheumatoid arthritis, reported in the GWAS catalog, at the conventional threshold of *P* < 5 × 10^−8^ within 200 kb of the transcription site of the target gene. As *cis*‐SNPs were excluded from the GATE score, this is orthogonal validation. The 200‐kb cutoff is based on the distribution of distances from protein QTL variants to transcription start site.^16^ Although this criterion would not be appropriate for identifying the gene that mediates the *cis* effect of a disease‐associated SNP, if the gene has already been identified through *trans* effects as a putative core gene for the disease and there is a SNP association near its transcription site, then this gene is the most likely mediator of the *cis* effect.
2. Instrumental variable analysis (“Mendelian randomization”), based on marginalizing over the distribution of pleiotropic effects of *trans*‐QTLs, supports a causal effect of the transcript or protein at *P* < 0.01. This criterion is evaluated only if the target gene has at least 10 *trans*‐QTLs because, otherwise, there is not enough information to infer the distribution of pleiotropic effects.
3. Association of disease with the measured level of the encoded protein in the UK Biobank proteomics study, in the same direction as the association with the aggregated *trans* score, with magnitude (standardized log odds ratio) of the protein association at least twice that of the *trans* score association. Evidence of causality is strengthened if levels of the protein are associated with incident rheumatoid arthritis, excluding those already diagnosed at baseline.
4. Drugs targeting the gene product, its ligand, or its receptor cause inflammatory arthritis or have shown efficacy against rheumatoid arthritis in a phase 2 trial.
5. Perturbation of the gene by knockout, transduction, or overexpression, or perturbation of the gene product by an inhibitor or an agonist, alters the severity of disease in an experimental model of inflammatory arthritis.
6. Rare variants in the gene cause a monogenic form of the disease. From searching PubMed, 11 genes were identified as reported monogenic causes of inflammatory arthritis: *LACC1*, *LRBA*, *NFIL3*, *UNC13D*, *NOD2*, *NLRP3*, *MEFV*, *TNFAIP3*, *PRF1*, *STX11*, *ACP5*.^17^, ^18^, ^19^, ^20^, ^21^

## RESULTS

### *Trans*‐eQTL scores

Table 1 shows the six putative core genes identified through association with aggregated *trans*‐eQTL scores. For three of these genes—*CD5*, *CTLA4*, and *SLAMF1*—associations of rheumatoid arthritis with SNPs within 200 kb of the transcription site are listed in the GWAS catalog. For these SNP associations, gene names recorded in the GWAS catalog in the fields “Reported Gene” or “Mapped Gene” are shown in the last column of Table 1. As previously reported for type 1 diabetes,^8^ the *cis*‐eQTL association of *CTLA4* is in the opposite direction to the association with the aggregated *trans* score; this may be explained by *cis*‐acting SNPs that alter the splicing of this gene.

**Table 1:** Putative core genes identified through aggregated *trans*‐eQTL scores*

	Transcription site		*Trans* score			*Cis* score		
Gene	Chrom	Start position (Mb)	Effective number of *trans*‐eQTLs	Log odds ratio	*P*	Log odds ratio	*P*	Reported GWAS hit within 200 kb
*IL10RA*	11	117.99	5.8	0.064	3 *×* 10^*−*6^	−0.015	0.3	
*CD5*	11	61.10	8.9	0.066	2 *×* 10^*−*6^	0.033	0.02	*CD6, CD5, VPS37C*
*CTLA4*	2	203.85	9.6	0.112	6 *×* 10^*−*16^	−0.037	0.007	*CD28, CTLA4, ICOS*
*STAP1*	4	67.56	6.4	−0.070	4 *×* 10^*−*7^	−0.014	0.3	
*FBLN7*	2	112.14	7.5	0.068	3 *×* 10^*−*6^	−0.008	0.6	
*SLAMF1*	1	160.61	5.7	0.070	4 *×* 10^*−*7^	0.009	0.5	*SLAMF6*

* Log odds ratios are scaled as the difference in log odds associated with a score difference of one SD. Threshold for inclusion in this table is *trans* score association at *P <* 10^*−*5^ and effective number of *trans*‐eQTLs >5. Chrom, chromosome; eQTL, expression quantitative trait locus; GWAS, genome‐wide association study; Mb, megabase.

**Figure 2:**
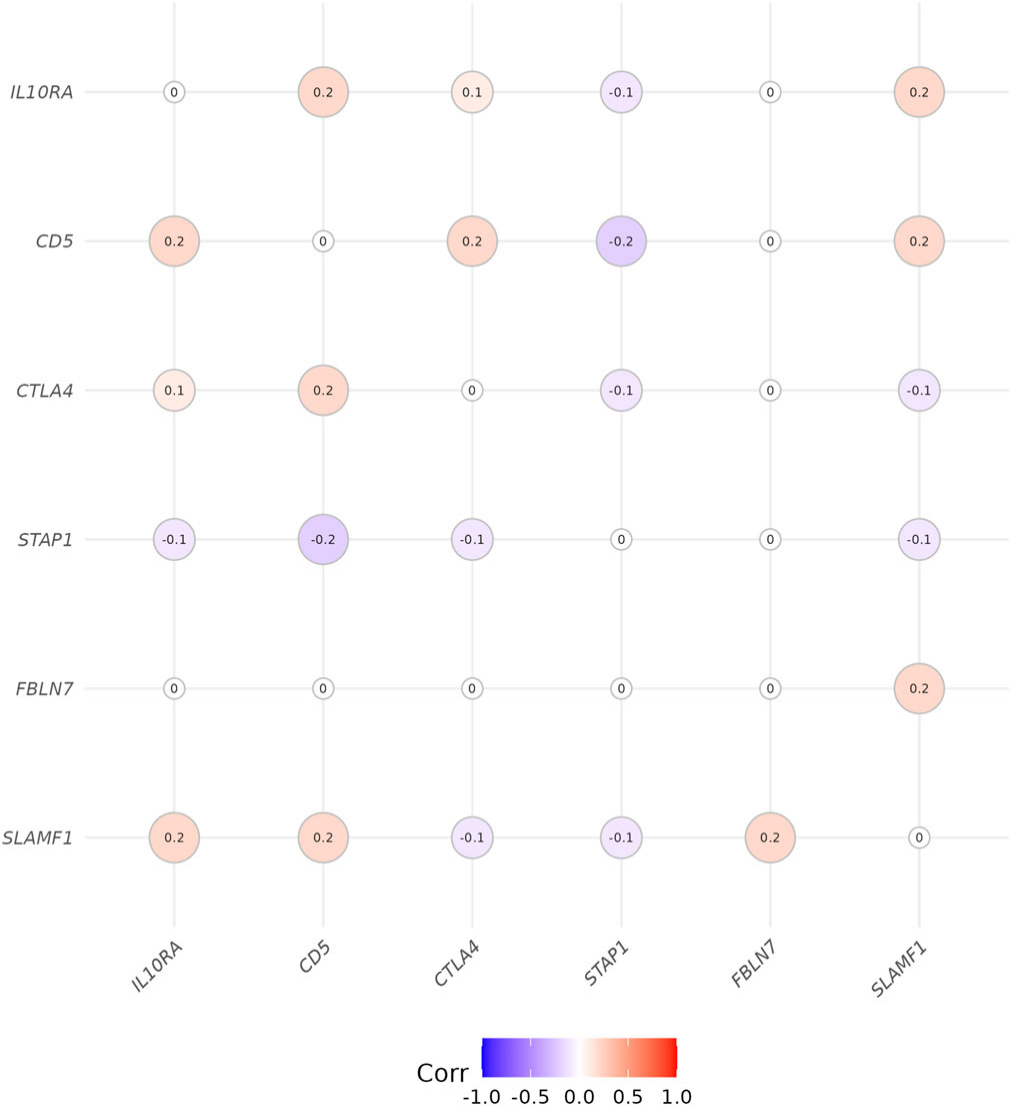
Correlations between aggregated *trans*‐eQTL scores for putative core genes. Rows and columns of the correlation matrix are ordered by hierarchical clustering on the absolute value of correlation. Diagonal elements of the correlation matrix are equal to one; they are set to zero for easier visualisation of the off‐diagonal values. eQTL, expression quantitative trait locus. Color figure can be viewed in the online issue, which is available at http://onlinelibrary.wiley.com/doi/10.1002/art.43125/abstract.

Figure 2 shows that the aggregated *trans*‐eQTL scores for the six putative core genes are only weakly correlated, indicating that they do not share most of their *trans*‐eQTLs. Supplementary Table S1 shows that multiple regions containing SNPs previously identified as associated with rheumatoid arthritis are *trans*‐eQTLs for one or more of these putative core genes. Notably, seven regions outside the HLA region that contain SNPs associated with rheumatoid arthritis are *trans*‐eQTLs for *CTLA4*.

To test whether genetic effects on the proportions of immune cell types could explain the associations of rheumatoid arthritis with *trans* scores for predicted gene expression, we used summary statistics from the SardiNIA study of immune cell phenotypes in peripheral blood^22^ to calculate genome‐wide scores for each immune cell phenotype. We tested these scores for association with rheumatoid arthritis. Supplementary Table S2 shows that rheumatoid arthritis was associated inversely with the proportion of CD4+ T cells that were Treg cells and positively associated with the absolute count of CD4+ T cells. Although *CTLA4* is expressed on CD4+ T cells, the association of rheumatoid arthritis with the polygenic score for CD4+ T cell count was much weaker than the association with the GATE score for *CTLA4*.

### *Trans*‐pQTL scores

Table 2 shows the 10 putative core genes identified through association with aggregated *trans*‐pQTL scores. The strongest GATE score association with rheumatoid arthritis is for *PDCD1*, which encodes the immune checkpoint receptor programmed cell death protein 1 (PD‐1). Supplementary Table S3 shows that the *trans*‐pQTLs contributing to this GATE score include 12 regions containing genes previously associated with rheumatoid arthritis through GWAS studies: *SDF4, AP4B1, PTPN22, CTLA4, BACH2, TRAF1, IL2RA, TSPAN14, PTPN11, ILF3, FUT2* and *AIRE*. This coalescence of *trans* effects of disease‐associated SNPs on expression of a disease‐relevant target gene is consistent with the sparse effector hypothesis.

**Table 2:** Putative core genes identified through aggregated *trans*‐pQTL scores*

			Transcription site		*Trans* score			*Cis* score		
pQTL study	N	Gene	Chrom	Start position (Mb)	Effective number of *trans*‐pQTLs	Log odds ratio	*P* value	Log odds ratio	*P* value	Reported GWAS hit within 200 kb
DeC	5,292	*PNLIPRP2*	10	116.62	6.2	0.061	9 *×* 10^*−*6^	−0.007	0.6	
UKB	4,650	*TP53BP1*	15	43.40	8.3	0.072	8 *×* 10^*−*7^			
UKB	4,650	*CXCL10*	4	76.02	7.6	0.091	1 *×* 10^*−*9^	0.019	0.2	
UKB	4,650	*CXCL9*	4	76.00	7.8	0.086	3 *×* 10^*−*9^	−0.007	0.6	
UKB	4,650	*IDO1*	8	39.90	8.2	0.065	9 *×* 10^*−*6^	−0.025	0.1	
UKB	4,650	*PDCD1*	2	241.85	21.3	0.096	1 *×* 10^*−*10^	−0.005	0.7	*GAL3ST2*
UKB	4,650	*TNFRSF14*	1	2.56	9.8	0.067	4 *×* 10^*−*6^	−0.024	0.1	*TNFRSF14, MMEL1*
UKB	4,650	*LAIR1*	19	54.35	6.1	0.069	4 *×* 10^*−*6^	−0.006	0.7	
DeC	5,292	*TIGIT*	3	114.28	6.1	0.082	2 *×* 10^*−*9^	0.004	0.8	
DeC	5,292	*LILRA4*	19	54.33	9.1	0.063	5 *×* 10^−6^	−0.009	0.5	

* Log odds ratios are scaled as the difference in log odds associated with a score difference of one SD. Threshold for inclusion in this table is *trans* score association at *P <* 10^*−*5^ and effective number of *trans*‐pQTLs >5. Chrom, chromosome; DeC, deCODE; GWAS, genome‐wide association study; Mb, megabase; pQTL, protein quantitative trait locus; UKB, UK Biobank.

Supplementary Figure S1 shows that the aggregated *trans*‐pQTL scores for seven of these putative core genes are correlated, indicating that they share *trans*‐pQTLs. The highest correlation (0.6) is between the scores for *CXCL10* and *CXCL9*. Supplementary Table S3 shows that these two genes share nine *trans*‐pQTL regions containing genes—*PTPN22*, *GCKR, IL18R1, STAT4, CCR1, IFNG‐AS1, PTPN11*, *PTPN2*, and *TYK2*—previously identified as associated with rheumatoid arthritis. This sharing of *trans*‐QTLs means that we cannot distinguish, without other evidence, which of these two target genes is most likely to be causal.

To investigate whether these results were replicated in independent datasets, we searched the “expression quantitative trait score” (eQTS) summary statistics released by the eQTLGen Consortium.^9^ Their study used summary statistics from two published GWAS of rheumatoid arthritis to construct polygenic risk scores that were tested for association with gene expression in an individual‐level dataset. Supplementary Table S4 shows that for three of the genes in Tables 1 or 2—*PDCD1*, *TIGIT*, and *CTLA4*—expression levels were associated with polygenic scores for rheumatoid arthritis.

### Nearby GWAS hits

For five of the 16 putative core genes—*CD5*, *CTLA4*, *SLAMF1*, *PDCD1*, and *TNFRSF14*—that were identified through association of GATE scores with rheumatoid arthritis, associations of rheumatoid arthritis with SNPs within 200 kb of the transcription site are listed in the GWAS catalog. The attribution of the GWAS hit to the target gene is supported by a *cis*‐QTL association for *CTLA4*. For *CD5*, *CTLA4*, and *TNFRSF14*, the target gene is included in the GWAS catalog as one of the attributed genes. The association with SNPs in the *SLAMF1* region had been attributed to *SLAMF6*, and the association with SNPs in the *PDCD1* region had been attributed to *GAL3ST2*, 40 kb upstream of *PDCD1*.

### Mendelian randomization

Supplementary Table S5 shows that of the 10 putative core genes identified through aggregated *trans* effects on protein levels, six were supported at *P* < 0.01 by a Mendelian randomization analysis based on computing the marginal likelihood of the causal effect parameter.^15^ Scatter plots of the coefficients for the effects of the *trans*‐pQTLs are shown for *LAIR1*, *TP53BP1*, *TNFRSF14*, and *PDCD1* in Supplementary Figures S2–S5. The coefficients for the effects of the pQTLs on rheumatoid arthritis are plotted against the coefficients for the effects of the pQTLs on circulating levels of the protein. The slope of the straight line shown in each plot is the maximum likelihood estimate of the causal effect parameter based on marginalizing over the posterior distribution of the direct (pleiotropic) effects. If there were no direct effects of the *trans*‐pQTLs on rheumatoid arthritis and no uncertainty in the estimates of coefficients of regression of disease and protein levels on the *trans*‐pQTLs, the points in this plot would lie on a straight line through the origin, and the slope of this line would be the causal effect parameter. We emphasize that Mendelian randomization analysis has limitations and that the results should be interpreted as only one line of evidence to be combined with evidence from other sources.

### Associations with measured levels of proteins and transcripts

Of the 54,306 individuals in the UK Biobank proteomics study, 688 had a diagnosis of primary rheumatoid arthritis at baseline or during follow‐up. Table 3 shows that of the 14 putative core genes for which the protein encoded by the gene was measured in the UK Biobank proteomics study, nine met the criterion of association in the same direction as the *trans* score effect with a standardized log odds ratio at least twice as high for the measured protein as for the GATE score that predicts the protein level. Because these associations of protein levels with rheumatoid arthritis include cases already diagnosed, they may be altered by the effects of the disease or by drug treatment.

**Table 3:** Tests of association of rheumatoid arthritis (at baseline or follow‐up) with plasma levels of proteins measured in the UK Biobank cohort and encoded by putative core genes or by genes within 200 kb of a reported GWAS hit*

		Measured level of protein				*Trans* score				
Gene	GWAS hit	Noncases	Cases	Log odds ratio	*P* value	Effective number of *trans*‐pQTLS	*r* ^2^	Ratio *r* ^2^ / *h* ^2^_*trans*_	Log odds ratio	*P* value
Genes in Tables 1 or 2 with protein levels measured in UK Biobank										
*PDCD1*	*GAL3ST2*	50,779	698	0.60	2 × 10^−65^	21.3	0.034	0.17	0.10	1 × 10^−10^
*LAIR1*		50,426	684	0.47	4 × 10^−45^	6.1	0.006	0.04	0.07	4 × 10^−6^
*CXCL10*		50,674	688	0.45	1 × 10^−31^	7.6	0.015	0.16	0.09	1 × 10^−9^
*TNFRSF14*	*TNFRSF14, MMEL1*	50,507	686	0.43	3 × 10^−29^	9.8	0.010	0.07	0.07	4 × 10^−6^
*CXCL9*		50,674	688	0.37	7 × 10^−21^	7.8	0.015	0.12	0.09	3 × 10^−9^
*SLAMF1*	*SLAMF6*	49,524	677	0.24	1 × 10^−10^	10.3	0.013	0.15	0.05	3 × 10^−4^
*TIGIT*		43,025	589	0.22	1 × 10^−8^	4.0	0.004	0.24	0.01	0.6
*CD5*	*CD6, CD5, VPS37C*	50,790	698	0.17	6 × 10^−6^	11.3	0.022	0.13	0.04	0.005
*IL10RA*		49,524	677	0.12	0.001	3.9	0.001	1.55	0.03	0.05
*TP53BP1*		42,549	583	0.13	0.001	8.3	0.022	0.25	0.07	8 × 10^−7^
*CTLA4*		43,828	598	−0.12	0.003					
*PNLIPRP2*		50,426	684	−0.05	0.2	5.4	0.004	0.04	0.04	0.007
*LILRA4*		42,371	589	0.04	0.3	7.0	0.003		0.01	0.7
*IDO1*		42,660	584	−0.02	0.6	7.8	0.020	0.15	0.05	2 × 10^−4^
Genes within 200 kb of a GWAS hit and *trans* score association with rheumatoid arthritis at *P <* 0.001										
*LGALS9*	*KSR1, NOS2*	50,431	685	0.64	9 × 10^−64^	8.6	0.015	0.10	0.06	3 × 10^−5^
*IL2RA*	*IL2RA*	50,102	681	0.51	3 × 10^−42^	11.6	0.016	0.09	0.06	9 × 10^−5^
*TNFRSF9*	*TNFRSF9, PARK7*	50,067	683	0.46	4 × 10^−38^	9.0	0.017	0.09	0.06	1 × 10^−4^
*CD274*	+	50,067	683	0.46	5 × 10^−36^	8.3	0.017	0.16	0.05	5 × 10^−4^
*CD27*	*TNFRSF1A, LTBR*	50,608	687	0.44	8 × 10^−35^	24.2	0.021	0.11	0.06	1 × 10^−4^
*CD48*	*ITLN1*	50,590	688	0.21	7 × 10^−8^	15.6	0.035	0.17	0.06	1 × 10^−4^
*SLAMF7*	*ITLN1*	50,386	684	0.06	0.1	20.5	0.035	0.22	0.05	9 × 10^−4^
*GP1BB*	*SEPT5‐GP1BB, TBX1*	42371	589	−0.02	0.6	9.8	0.041	0.25	0.05	3 × 10^−4^

* Associations are adjusted for age, sex, and continental ancestry. Log odds ratios are scaled as difference in log odds associated with a covariate difference of one SD. *Trans* scores are based on proteins measured in UK Biobank only. For *TIGIT*, *PNLIPR2*, and *LILRA4*, these associations differ from those in Table 2, which are based on *trans*‐pQTLs for proteins measured in the DeCODE study. Similarly, for *CD5*, *IL10RA*, and *SLAMF1*, these differ from those in Table 1, which are based on *trans*‐eQTLs for transcripts measured in the eQTLGen study. The plus sign denotes GWAS hits that have not been attributed to a gene. GWAS, genome‐wide association study; pQTL, protein quantitative trait locus.

The lower part of Table 3 shows eight additional candidates for core gene status identified as genes within 200 kb of a GWAS hit that have a GATE score associated with rheumatoid arthritis at the less stringent threshold of *P* < 0.001 (on the basis that a nearby GWAS hit increases the prior probability of a causal effect). For five of these eight genes—*LGALS9*, *IL2RA*, *TNFRSF9*, *CD274*, and *CD27*—levels of the encoded protein were strongly associated with rheumatoid arthritis. Supplementary Table S6 shows that for 10 of the 14 proteins associated with rheumatoid arthritis at baseline or follow‐up at a standardized log odds ratio >0.2, these associations persisted after restriction to incident cases diagnosed during follow‐up.

Table 3 shows that the squared correlations (*r* ^2^) between the GATE scores and the measured protein levels are typically only about 2% and that the proportion of variance explained by the GATE score is typically 10% to 20% of the total SNP heritability attributable to *trans* effects (*h* ^2^_*trans*_) estimated previously in the UK Biobank proteomics dataset.^12^ If the association of the protein with disease is causal, the effect size associated with the measured protein level should be about seven times the effect size associated with the GATE score (the ratio of effect sizes scales with the correlation of the score with the measured value). For the top five genes in Table 3, the ratio of measured protein effect size to GATE score effect size is between four and seven, broadly consistent with this prediction.

To compare whole blood transcript levels in cases of rheumatoid arthritis and healthy controls, we examined two microarray studies: a published study of 66 cases of rheumatoid arthritis not yet treated with a conventional synthetic disease‐modifying antirheumatic drug (csDMARD) and 35 healthy controls in Keio, Japan^23^ and an unpublished study of 55 csDMARD‐treated cases of rheumatoid arthritis and 10 healthy controls in Manchester, England. Supplementary Table S7 shows that expression levels of *CD5* and *LAIR1* were inversely associated with rheumatoid arthritis in both studies. These associations with transcript levels were opposite in direction to the associations with the soluble proteins in UK Biobank, consistent with the possibility that the soluble proteins act as decoys to down‐regulate the cellular receptor. We emphasize that to characterize case‐control differences in whole blood transcript levels will require larger studies using methods that distinguish splice variants and control for cell‐type proportions.

### Summary of validation

Supplementary Table S8 tabulates the genes in Tables 1, 2, or 3 for which experimental perturbation has demonstrated an effect in a mouse model of inflammatory arthritis. For two of these genes, there is at least preliminary clinical evidence of efficacy of drugs targeting the encoded protein against rheumatoid arthritis. Table 4 summarizes the results of applying five criteria for validation of each putative core gene, excluding the sixth criterion of monogenic disease, which was not met by any of these genes. The strongest evidence is for *PDCD1*, which meets all five criteria: nearby GWAS hit, protein association, Mendelian randomization support, validation from perturbation in an experimental model, and effects of a targeted agonist against rheumatoid arthritis in a phase 2 trial.

**Table 4:** Summary of validation status of putative core genes for rheumatoid arthritis identified through GATE analysis*

Gene	GWAS hit	Protein association	Mendelian randomization	Experimental validation in mouse model	Drug effect in humans
*IL10RA*				+	
*CD5*	+	+		+	
*CTLA4*	+			+	+
*STAP1*					
*FBLN7*					
*SLAMF1*	+	+			
*PNLIPRP2*					
*TP53BP1*			+	+	
*CXCL10*		+		+	
*CXCL9*		+		+	
*IDO1*			+		
*PDCD1*	+	+	+	+	+
*TNFRSF14*	+	+	+	+	
*LAIR1*		+	+	+	
*TIGIT*		+		+	
*LILRA4*			+		

* GATE, genome‐wide aggregated *trans* effects; GWAS, genome‐wide association study.

## DISCUSSION

From aggregated *trans* effects, this study identified 16 genes outside the HLA region as putative core genes for rheumatoid arthritis. Six of these—*CD5*, *CTLA4*, *TIGIT*, *LAIR1*, *PDCD1*, and *TNFRSF14*—encode proteins that have been identified as immune checkpoints, defined broadly as receptors on immune cells that are exploited by cancer cells to escape the immune response.^24^, ^25^, ^26^, ^27^ Inflammatory arthritis and other autoimmune reactions are common adverse effects of inhibitors of these immune checkpoints used in cancer therapy. *LILRA4* encodes leukocyte immunoglobin‐like receptor subfamily A member 4, a cell surface protein expressed on plasmacytoid dendritic cells; it has been proposed that *LILRA4* is an “innate checkpoint,” analogous to the checkpoints identified in the adaptive immune system.^28^

For 10 of these genes, experimental perturbation in a mouse model has been shown to influence inflammatory arthritis.^29^, ^30^, ^31^, ^32^, ^33^, ^34^, ^35^, ^36^, ^37^, ^38^ For two putative core genes—*CTLA4* and *PDCD1*—there is at least preliminary evidence of efficacy against rheumatoid arthritis in clinical trials of drugs that target the protein.^39^, ^40^

Of the six immune checkpoint genes identified through GATE analysis as putative core genes for rheumatoid arthritis, GATE scores for four—*CD5*, *CTLA4*, *PDCD1*, and *TIGIT*—are associated with type 1 diabetes also^8^ (result for *PDCD1* to be reported elsewhere). The other two—*LAIR1* and *TNFRSF14*—appear to be more specifically associated with rheumatoid arthritis. *TNFRSF14* was originally identified as a receptor for glycoprotein D of herpes simplex virus; it is now considered to be an immune checkpoint that inhibits immune response by binding to B‐ and T‐lymphocyte attenuator, encoded by *BTLA*, and to CD160, an Ig‐like glycoprotein expressed on γδ T cells. A TNFRSF14‐Ig fusion protein aggravated autoimmune arthritis in the collagen‐induced arthritis mouse model.^34^

Of the 16 putative core genes identified through GATE analysis, 11 are expressed specifically by immune cells, mostly T cells. The genes not expressed specifically by immune cells are *IDO1*, *FBLN7*, *STAP1*, *TP53BP1*, and *PNLIPRP2*. *IDO1* modulates immune checkpoint–mediated immunosuppression by mechanisms that are not yet clear.^41^ *STAP1* has been reported to up‐regulate activation of T cells via the T cell receptor.^42^ For *FBLN7* and *TP53BP1*, there is some experimental support for a role in inflammatory arthritis. *FBLN7* encodes the extracellular matrix protein fibulin‐7, which binds to human monocytes via integrins *α*_5_ *β*_1_ and *α*_2_ *β*_1_; in a mouse model of systemic inflammation, it inhibits chemokine‐mediated migration of macrophages.^43^ *TP53BP1* encodes the p53‐binding protein 1 that is required for accumulation of p53 to facilitate repair of DNA double‐strand breaks. Knockout of the gene encoding p53 in mice increases disease severity in the collagen‐induced arthritis model.^32^ *PNLIPRP2*, which is only just above the *P*‐value threshold of 10^−5^ and has no support from association of measured protein levels with disease, may be a chance finding.

Six additional candidates for core gene status are identified by the combination of a GATE score association with rheumatoid arthritis at *P* < 0.001, a reported GWAS hit within 200 kb of the transcription site, and association of measured protein levels with rheumatoid arthritis. For five of these genes—*LGALS9*, *IL2RA*, *TNFRSF9*, *CD274*, and *CD27*—there is experimental validation from perturbation in a mouse model of inflammatory arthritis.^38^, ^44^, ^45^, ^46^, ^47^ For three of these five genes, the GWAS hit had been attributed to another nearby gene or not attributed at all. The association with *CD274* that encodes the ligand for the PD‐1 receptor is further support for a key role of PD‐1 signaling in rheumatoid arthritis. *LGALS9* encodes galectin‐9, one of four ligands for the immune checkpoint receptor TIM‐3, also known as hepatitis A virus cellular receptor 2; blockade of TIM‐3 ameliorates arthritis in a mouse model.^48^

The *trans*‐pQTL scores calculated in this study are for the predicted level of the circulating protein. In cases where this protein is the soluble form of a cellular receptor, the associations of disease with levels of the soluble form are not necessarily in the same direction as the associations with expression of the receptor. Thus, for PD‐1, the measured level of the soluble protein and the GATE score for the protein level are positively associated with rheumatoid arthritis. This effect is in the opposite direction to what would be predicted from clinical experience with antagonists and agonists of the PD‐1 receptor. A possible explanation for this is that the soluble protein acts as a decoy for the ligand for the cellular receptor.^49^, ^50^

Strengths of this study are that the cases and non‐cases are from a single cohort, that diagnoses based on primary care records or self‐report are validated by drug prescriptions, and that the design effectively rules out reverse causation because the background prevalence of rheumatoid arthritis in the population samples from which genetic scores were constructed was only about 1%. The main limitation of this study is that for genetic prediction from *trans* effects on gene expression in whole blood, it relies on eQTLGen phase 1, in which only 10,316 trait‐associated SNPs were tested for *trans* associations. Another limitation is that the eQTLGen summary statistics were not directly adjusted for cell‐type proportions in each blood sample, although they were adjusted for principal components as a proxy for cell‐type proportions. We addressed this by examining the associations of rheumatoid arthritis with polygenic scores for immune cell phenotypes; CD4+ T cells were the only cell type for which a polygenic score was positively associated with rheumatoid arthritis. As the GATE score associations with rheumatoid arthritis do not match the expression profile of this cell type, it is unlikely that confounding by cell‐type proportions can explain these associations. These limitations will be overcome when more comprehensive summary statistics are available from the whole blood transcriptomics study that is now planned for a subset of the UK Biobank cohort. For *trans*‐pQTLs in whole blood, comprehensive summary statistics on SNP associations are available for 2,923 proteins on the Olink platform measured on 54,000 participants in UK Biobank, but even with this large sample size, the GATE scores typically explain only about 10% of the estimated SNP heritability attributable to *trans* effects. We are nevertheless able to detect associations of these GATE scores with disease because the underlying associations, estimated from the associations of measured protein levels with disease, are typically strong.

Where variants with pleiotropic effects on gene expression have large effects on disease risk, GATE analysis alone cannot reliably distinguish causal genes from genes that are regulated by these pleiotropic variants. For this reason, we excluded *trans*‐QTLs in the HLA region from the computation of GATE scores, as in our earlier study of type 1 diabetes. Where variants have large direct effects on disease risk, the eQTS method^9^ is expected to be less powerful than GATE analysis because such variants dilute the association of the target gene with the polygenic risk score. This may explain why only three of the 16 putative core genes were replicated in the eQTS summary statistics, even though the eQTS analysis was not limited to the 10,316 SNPs used in eQTLGen phase 1.

The coalescence of disease‐associated SNP effects on expression of a small number of target genes, demonstrated in this study, provides broad support for the sparse effector hypothesis, at least for immune‐mediated inflammatory disease. As disease‐relevant genes are enriched with redundant enhancer domains and depleted of *cis*‐eQTLs of large effect,^51^, ^52^ they are often not detected by a conventional SNP‐by‐SNP GWAS analysis. Whatever theoretical questions remain to be resolved, it is clear that GATE analysis identifies disease‐relevant genes that include at least some promising therapeutic targets, in contrast with conventional SNP‐by‐SNP analysis of a GWAS, which identifies mostly SNPs of tiny effect near genes that often have no obvious relevance to disease‐specific pathways. Because GATE scores typically account for only a small proportion of the variance of gene expression, they are unlikely to be useful for stratifying disease with respect to prognosis and prediction of drug response. The proteins and transcripts encoded by the genes that are identified by GATE analysis as core genes have much stronger associations with disease; establishing whether they are useful as clinical predictors will require validation studies in cohorts.

## AUTHOR CONTRIBUTIONS

All authors contributed to at least one of the following manuscript preparation roles: conceptualization AND/OR methodology, software, investigation, formal analysis, data curation, visualization, and validation AND drafting or reviewing/editing the final draft. As corresponding author, Dr McKeigue confirms that all authors have provided the final approval of the version to be published and takes responsibility for the affirmations regarding article submission (eg, not under consideration by another journal), the integrity of the data presented, and the statements regarding compliance with institutional review board/Declaration of Helsinki requirements.

## Supporting information


**Disclosure Form**:

**Appendix S1:** Supporting Information

## References

[1] McKeigue P. Quantifying performance of a diagnostic test as the expected information for discrimination: relation to the C‐statistic. Stat Methods Med Res 2019;28(6):1841–1851. 29978758 10.1177/0962280218776989

[2] Kuo C‐F , Grainge MJ , Valdes AM , et al. Familial aggregation of rheumatoid arthritis and co‐aggregation of autoimmune diseases in affected families: a nationwide population‐based study. Rheumatology (Oxford) 2017;56(6):928–933. 28160009 10.1093/rheumatology/kew500 PMC5850742

[3] Schaid DJ , Lin WY . One‐ and two‐locus models for mapping rheumatoid arthritis‐susceptibility genes on chromosome 6. BMC Proc 2007;1(Suppl 1):S103. 18466443 10.1186/1753-6561-1-s1-s103 PMC2367548

[4] Ishigaki K , Sakaue S , Terao C , et al; BioBank Japan Project . Multi‐ancestry genome‐wide association analyses identify novel genetic mechanisms in rheumatoid arthritis. Nat Genet 2022;54(11):1640–1651. 36333501 10.1038/s41588-022-01213-w PMC10165422

[5] Fang H , Chen L , Knight JC . From genome‐wide association studies to rational drug target prioritisation in inflammatory arthritis. Lancet Rheumatol 2020;2(1):e50–e62. 38258277 10.1016/S2665-9913(19)30134-1

[6] Liu X , Li YI , Pritchard JK . Trans effects on gene expression can drive omnigenic inheritance. Cell 2019;177(4):1022–1034.e6. 31051098 10.1016/j.cell.2019.04.014 PMC6553491

[7] Boyle EA , Li YI , Pritchard JK . An expanded view of complex traits: from polygenic to omnigenic. Cell 2017;169(7):1177–1186. 28622505 10.1016/j.cell.2017.05.038 PMC5536862

[8] Iakovliev A , McGurnaghan SJ , Hayward C , et al. Genome‐wide aggregated trans‐effects on risk of type 1 diabetes: a test of the “omnigenic” sparse effector hypothesis of complex trait genetics. Am J Hum Genet 2023;110(6):913–926. 37164005 10.1016/j.ajhg.2023.04.003 PMC10257008

[9] Võsa U , Claringbould A , Westra HJ , et al; BIOS Consortium; i2QTL Consortium . Large‐scale cis‐ and trans‐eQTL analyses identify thousands of genetic loci and polygenic scores that regulate blood gene expression. Nat Genet 2021;53(9):1300–1310. 34475573 10.1038/s41588-021-00913-z PMC8432599

[10] Sun BB , Maranville JC , Peters JE , et al. Genomic atlas of the human plasma proteome. Nature 2018;558(7708):73–79. 29875488 10.1038/s41586-018-0175-2 PMC6697541

[11] Ferkingstad E , Sulem P , Atlason BA , et al. Large‐scale integration of the plasma proteome with genetics and disease. Nat Genet 2021;53(12):1712–1721. 34857953 10.1038/s41588-021-00978-w

[12] Sun BB , Chiou J , Traylor M , et al; Alnylam Human Genetics; AstraZeneca Genomics Initiative; Biogen Biobank Team; Bristol Myers Squibb; Genentech Human Genetics; GlaxoSmithKline Genomic Sciences; Pfizer Integrative Biology ; Population Analytics of Janssen Data Sciences; Regeneron Genetics Center. Plasma proteomic associations with genetics and health in the UK Biobank. Nature 2023;622(7982):329–338. 37794186 10.1038/s41586-023-06592-6 PMC10567551

[13] Fehrmann RSN , Jansen RC , Veldink JH , et al. Trans‐eQTLs reveal that independent genetic variants associated with a complex phenotype converge on intermediate genes, with a major role for the HLA. PLoS Genet 2011;7(8):e1002197. 21829388 10.1371/journal.pgen.1002197 PMC3150446

[14] Hill MO . Diversity and evenness: a unifying notation and its consequences. Ecology 1973;54(2):427–432.

[15] McKeigue PM , Spiliopoulou A , Iakovliev A , et al. Inference of causal and pleiotropic effects with multiple weak genetic instruments: application to effect of adiponectin on type 2 diabetes. medRxiv Preprint posted online December 17, 2023. doi:10.1101/2023.12.15.23300008

[16] Fauman EB , Hyde C . An optimal variant to gene distance window derived from an empirical definition of cis and trans protein QTLs. BMC Bioinformatics 2022;23(1):169. 35527238 10.1186/s12859-022-04706-x PMC9082853

[17] Miceli‐Richard C , Lesage S , Rybojad M , et al. CARD15 mutations in Blau syndrome. Nat Genet 2001;29(1):19–20. 11528384 10.1038/ng720

[18] Diogo D , Kurreeman F , Stahl EA , et al; Consortium of Rheumatology Researchers of North America; Rheumatoid Arthritis Consortium International. Rare, low‐frequency, and common variants in the protein‐coding sequence of biological candidate genes from GWASs contribute to risk of rheumatoid arthritis. Am J Hum Genet 2013;92(1):15–27. 23261300 10.1016/j.ajhg.2012.11.012 PMC3542467

[19] Zhou Q , Wang H , Schwartz DM , et al. Loss‐of‐function mutations in TNFAIP3 leading to A20 haploinsufficiency cause an early‐onset autoinflammatory disease. Nat Genet 2016;48(1):67–73. 26642243 10.1038/ng.3459 PMC4777523

[20] McGonagle D , Watad A , Savic S . Mechanistic immunological based classification of rheumatoid arthritis. Autoimmun Rev 2018;17(11):1115–1123. 30213700 10.1016/j.autrev.2018.06.001

[21] La Bella S , Rinaldi M , Di Ludovico A , et al. Genetic background and molecular mechanisms of juvenile idiopathic arthritis. Int J Mol Sci 2023;24(3):1846. 36768167 10.3390/ijms24031846 PMC9916312

[22] Orrù V , Steri M , Sole G , et al. Genetic variants regulating immune cell levels in health and disease. Cell 2013;155(1):242–256. 24074872 10.1016/j.cell.2013.08.041 PMC5541764

[23] Tasaki S , Suzuki K , Kassai Y , et al. Multi‐omics monitoring of drug response in rheumatoid arthritis in pursuit of molecular remission. Nat Commun 2018;9(1):2755. 30013029 10.1038/s41467-018-05044-4 PMC6048065

[24] Ren S , Tian Q , Amar N , et al. The immune checkpoint, HVEM may contribute to immune escape in non‐small cell lung cancer lacking PD‐L1 expression. Lung Cancer 2018;125:115–120. 30429008 10.1016/j.lungcan.2018.09.004

[25] Ruth JH , Gurrea‐Rubio M , Athukorala KS , et al. CD6 is a target for cancer immunotherapy. JCI Insight 2021;6:e145662. 33497367 10.1172/jci.insight.145662 PMC8021120

[26] Helou DG , Shafiei‐Jahani P , Hurrell BP , et al. LAIR‐1 acts as an immune checkpoint on activated ILC2s and regulates the induction of airway hyperreactivity. J Allergy Clin Immunol 2022;149(1):223–236.e6. 34144112 10.1016/j.jaci.2021.05.042 PMC8674385

[27] Alotaibi FM , Min WP , Koropatnick J . CD5 blockade, a novel immune checkpoint inhibitor, enhances T cell anti‐tumour immunity and delays tumour growth in mice harbouring poorly immunogenic 4T1 breast tumour homografts. Front Immunol 2024;15:1256766. 38487537 10.3389/fimmu.2024.1256766 PMC10937348

[28] Tiberio L , Laffranchi M , Zucchi G , et al. Inhibitory receptors of plasmacytoid dendritic cells as possible targets for checkpoint blockade in cancer. Front Immunol 2024;15:1360291. 38504978 10.3389/fimmu.2024.1360291 PMC10948453

[29] Henningsson L , Eneljung T , Jirholt P , et al. Disease‐dependent local IL‐10 production ameliorates collagen induced arthritis in mice. PLoS One 2012;7(11):e49731. 23166758 10.1371/journal.pone.0049731 PMC3500327

[30] Miura Y , Isogai S , Maeda S , et al. CTLA‐4‐Ig internalizes CD80 in fibroblast‐like synoviocytes from chronic inflammatory arthritis mouse model. Sci Rep 2022;12(1):16363. 36180526 10.1038/s41598-022-20694-7 PMC9525600

[31] Plater‐Zyberk C , Taylor PC , Blaylock MG , et al. Anti‐CD5 therapy decreases severity of established disease in collagen type II‐induced arthritis in DBA/1 mice. Clin Exp Immunol 1994;98(3):442–447. 7527741 10.1111/j.1365-2249.1994.tb05510.x PMC1534513

[32] Simelyte E , Rosengren S , Boyle DL , et al. Regulation of arthritis by p53: critical role of adaptive immunity. Arthritis Rheum 2005;52(6):1876–1884. 15934085 10.1002/art.21099

[33] Zhao W , Dong Y , Wu C , et al. TIGIT overexpression diminishes the function of CD4 T cells and ameliorates the severity of rheumatoid arthritis in mouse models. Exp Cell Res 2016;340(1):132–138. 26683997 10.1016/j.yexcr.2015.12.002

[34] Pierer M , Schulz A , Rossol M , et al. Herpesvirus entry mediator‐Ig treatment during immunization aggravates rheumatoid arthritis in the collagen‐induced arthritis model. J Immunol 2009;182:3139–3145. 19234211 10.4049/jimmunol.0713715

[35] Kim S , Easterling ER , Price LC , et al. The role of leukocyte associated immunoglobulin‐like receptor‐1 (LAIR‐1) in suppressing collagen‐induced arthritis. J Immunol 2017;199:2692–2700. 28887430 10.4049/jimmunol.1700271 PMC5714324

[36] Kwak HB , Ha H , Kim HN , et al. Reciprocal cross‐talk between RANKL and interferon‐gamma‐inducible protein 10 is responsible for bone‐erosive experimental arthritis. Arthritis Rheum 2008;58(5):1332–1342. 18438854 10.1002/art.23372

[37] Boff D , Crijns H , Janssens R , et al. The chemokine fragment CXCL9(74‐103) diminishes neutrophil recruitment and joint inflammation in antigen‐induced arthritis. J Leukoc Biol 2018;104(2):413–422. 29733455 10.1002/JLB.3MA1217-502R

[38] Wood MK , Daoud A , Talor MV , et al. Programmed death ligand 1‐expressing macrophages and their protective role in the joint during arthritis. Arthritis Rheumatol 2024;76(4):553–565. 37997621 10.1002/art.42749 PMC12506893

[39] Maxwell L , Singh JA . Abatacept for rheumatoid arthritis. Cochrane Database Syst Rev 2009;2009(4):CD007277. 19821401 10.1002/14651858.CD007277.pub2 PMC6464777

[40] Tuttle J , Drescher E , Simón‐Campos JA , et al. A phase 2 trial of peresolimab for adults with rheumatoid arthritis. N Engl J Med 2023;388(20):1853–1862. 37195941 10.1056/NEJMoa2209856

[41] Zhai L , Ladomersky E , Lenzen A , et al. IDO1 in cancer: a Gemini of immune checkpoints. Cell Mol Immunol 2018;15(5):447–457. 29375124 10.1038/cmi.2017.143 PMC6068130

[42] Kagohashi K , Sasaki Y , Ozawa K , et al. Role of signal‐transducing adaptor protein‐1 for T cell activation and pathogenesis of autoimmune demyelination and airway inflammation. J Immunol 2024;212: 951–961. 38315039 10.4049/jimmunol.2300202

[43] Sarangi PP , Chakraborty P , Dash SP , et al. Cell adhesion protein fibulin‐7 and its C‐terminal fragment negatively regulate monocyte and macrophage migration and functions in vitro and in vivo. FASEB J 2018;32(9):4889–4898. 29634368 10.1096/fj.201700686RRR PMC6103172

[44] Seki M , Oomizu S , Sakata KM , et al. Galectin‐9 suppresses the generation of Th17, promotes the induction of regulatory T cells, and regulates experimental autoimmune arthritis. Clin Immunol 2008;127(1):78–88. 18282810 10.1016/j.clim.2008.01.006

[45] Sakaguchi S , Sakaguchi N , Asano M , et al. Immunologic self‐tolerance maintained by activated T cells expressing IL‐2 receptor alpha‐chains (CD25). Breakdown of a single mechanism of self‐tolerance causes various autoimmune diseases. J Immunol 1995;155:1151–1164. 7636184

[46] Seo SK , Choi JH , Kim YH , et al. 4‐1BB‐mediated immunotherapy of rheumatoid arthritis. Nat Med 2004;10(10):1088–1094. 15448685 10.1038/nm1107

[47] Oflazoglu E , Boursalian TE , Zeng W , et al. Blocking of CD27‐CD70 pathway by anti‐CD70 antibody ameliorates joint disease in murine collagen‐induced arthritis. J Immunol 2009;183:3770–3777. 19710474 10.4049/jimmunol.0901637

[48] Nozaki Y , Akiba H , Akazawa H , et al. Inhibition of the TIM‐1 and ‐3 signaling pathway ameliorates disease in a murine model of rheumatoid arthritis. Clin Exp Immunol 2024;218(1):55–64. 38975703 10.1093/cei/uxae056 PMC11404125

[49] Gu D , Ao X , Yang Y , et al. Soluble immune checkpoints in cancer: production, function and biological significance. J Immunother Cancer 2018;6(1):132. 30482248 10.1186/s40425-018-0449-0 PMC6260693

[50] Khan M , Zhao Z , Arooj S , et al. Soluble PD‐1: predictive, prognostic, and therapeutic value for cancer immunotherapy. Front Immunol 2020;11:587460. 33329567 10.3389/fimmu.2020.587460 PMC7710690

[51] Wang X , Goldstein DB . Enhancer domains predict gene pathogenicity and inform gene discovery in complex disease. Am J Hum Genet 2020;106(2):215–233. 32032514 10.1016/j.ajhg.2020.01.012 PMC7010980

[52] Mostafavi H , Spence JP , Naqvi S , et al. Systematic differences in discovery of genetic effects on gene expression and complex traits. Nat Genet 2023;55(11):1866–1875. 37857933 10.1038/s41588-023-01529-1 PMC12270542

