# Supplementary material for: Genome‐Wide Aggregated Trans Effects Analysis Identifies Genes Encoding Immune Checkpoints as Core Genes for Rheumatoid Arthritis
Source: Arthritis Rheumatol. 2025 Mar 16;77(7):817–26. doi: 10.1002/art.43125 (PMC12209750; doi:10.1002/art.43125)
Supplement: Supplementary file 2 — Appendix S1: Supporting Information [file ART-77-817-s001.pdf]

Genome-wide aggregated *trans*- effects analysis identifies genes encoding immune checkpoints as core genes for rheumatoid arthritis

Athina Spiliopoulou, Andrii Iakovliev, Darren Plant, Megan Sutcliffe, Seema Sharma, Cankut Cubuk, Myles Lewis, Costantino Pitzalis, Anne Barton, Paul M McKeigue

Supplementary information

Supplementary Figures

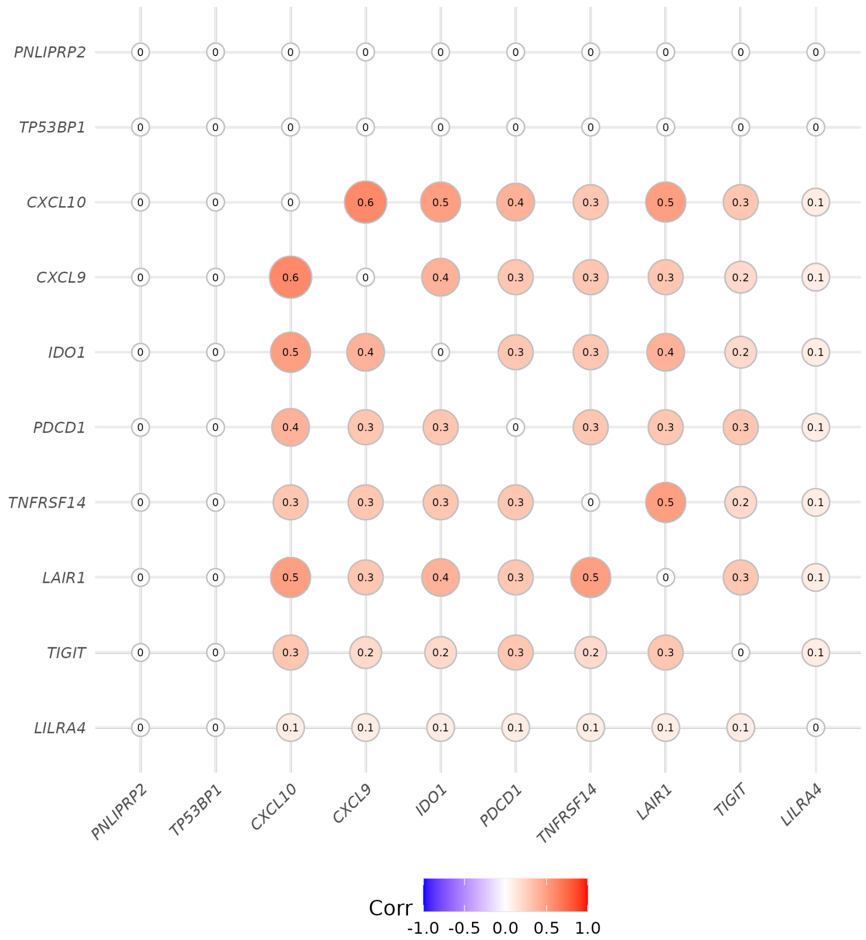

Fig S1. Correlations between aggregated pQTL scores for putative core genes. Rows and columns of correlation matrix are ordered by hierarchical clustering on absolute value of correlation

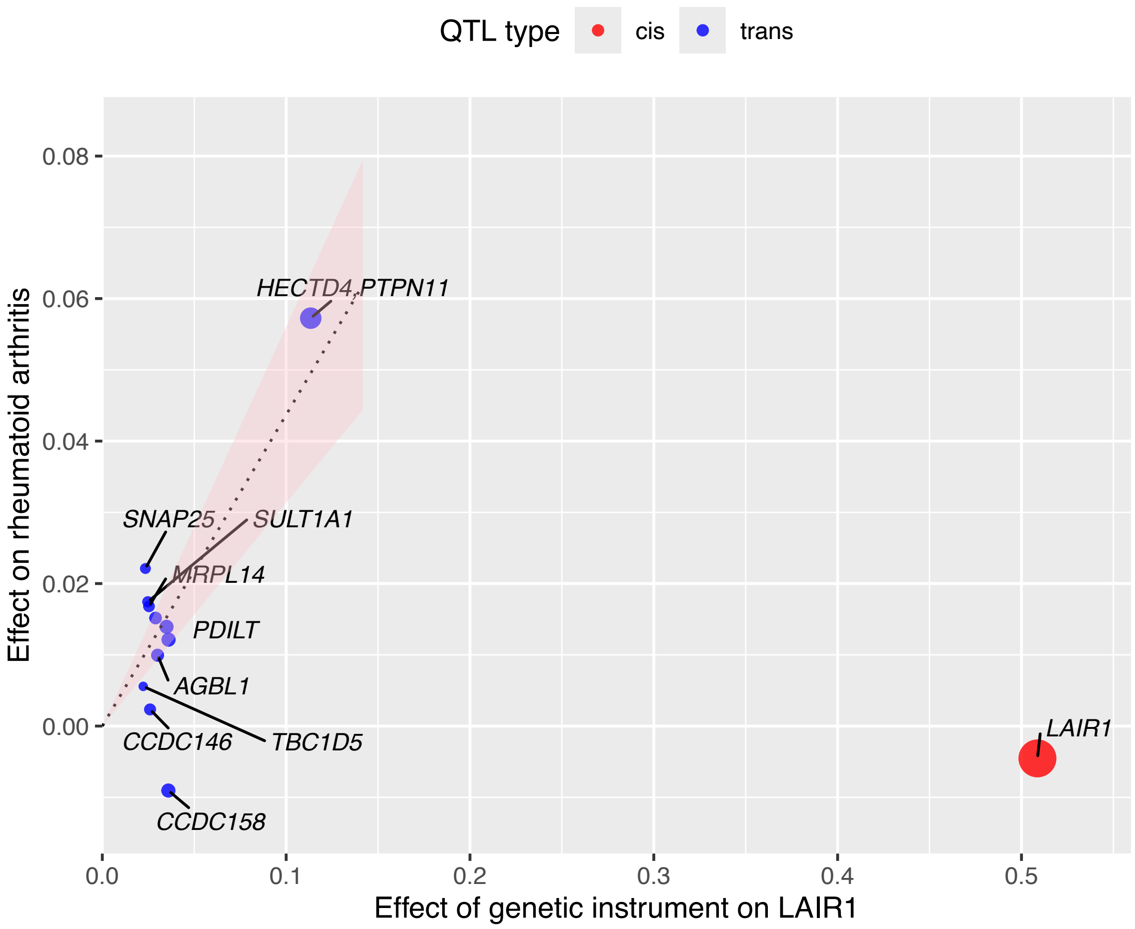

Fig S2. Mendelian randomization analysis of effect of *LAIR1* on rheumatoid arthritis. All *trans*-pQTLs with at least one SNP association with the protein at $p < 10^{-6}$ are included in the analysis, but the *cis*-pQTL (red circles) is excluded. The size of each point is inversely proportional to the standard error of the ratio estimator. The slope of the line is the maximum likelihood estimate of the causal effect parameter.

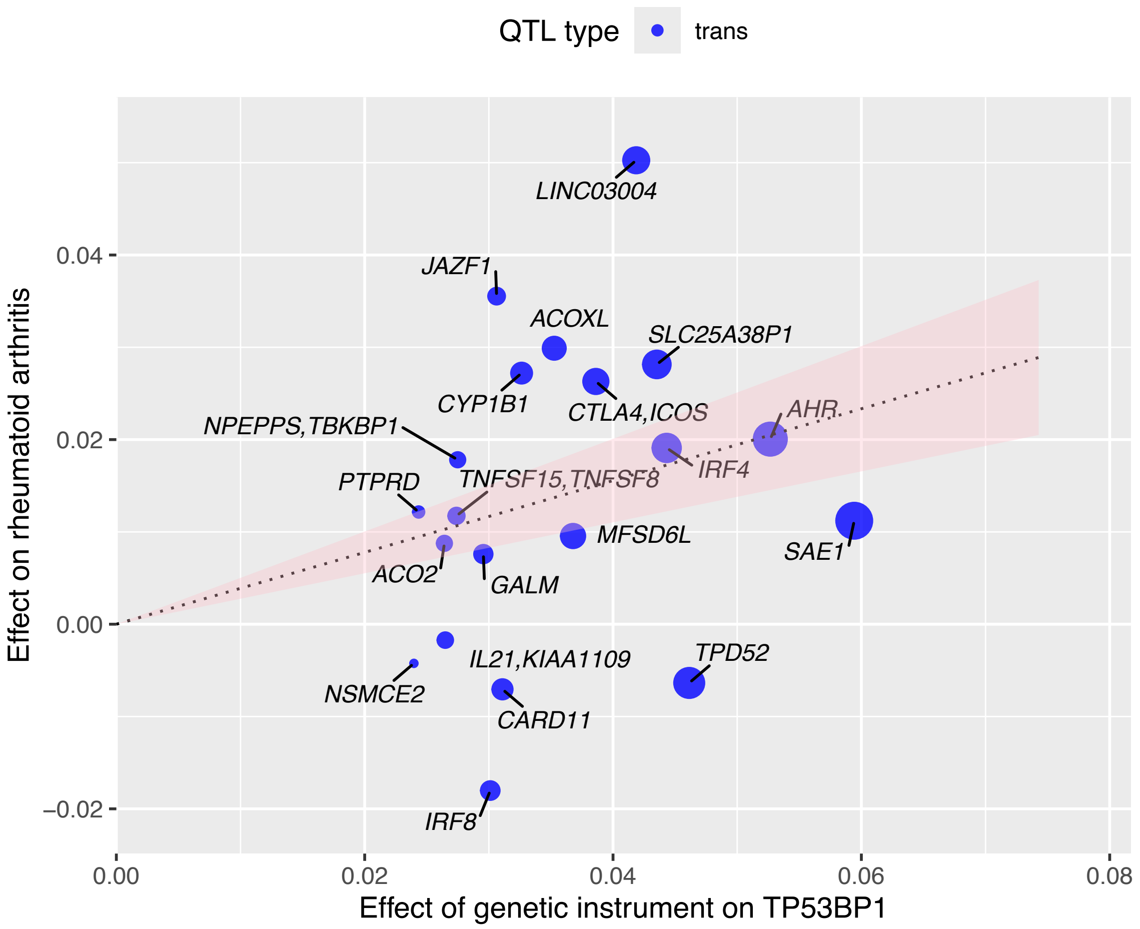

Fig S3. Mendelian randomization analysis of effect of *TP53BP1* on rheumatoid arthritis. All *trans*-pQTLs with at least one SNP association with the protein at $p < 10^{-6}$ are included in the analysis. The size of each point is inversely proportional to the standard error of the ratio estimator. The slope of the line is the maximum likelihood estimate of the causal effect parameter. The shaded ribbon is one standard error above and below the maximum likelihood estimate.

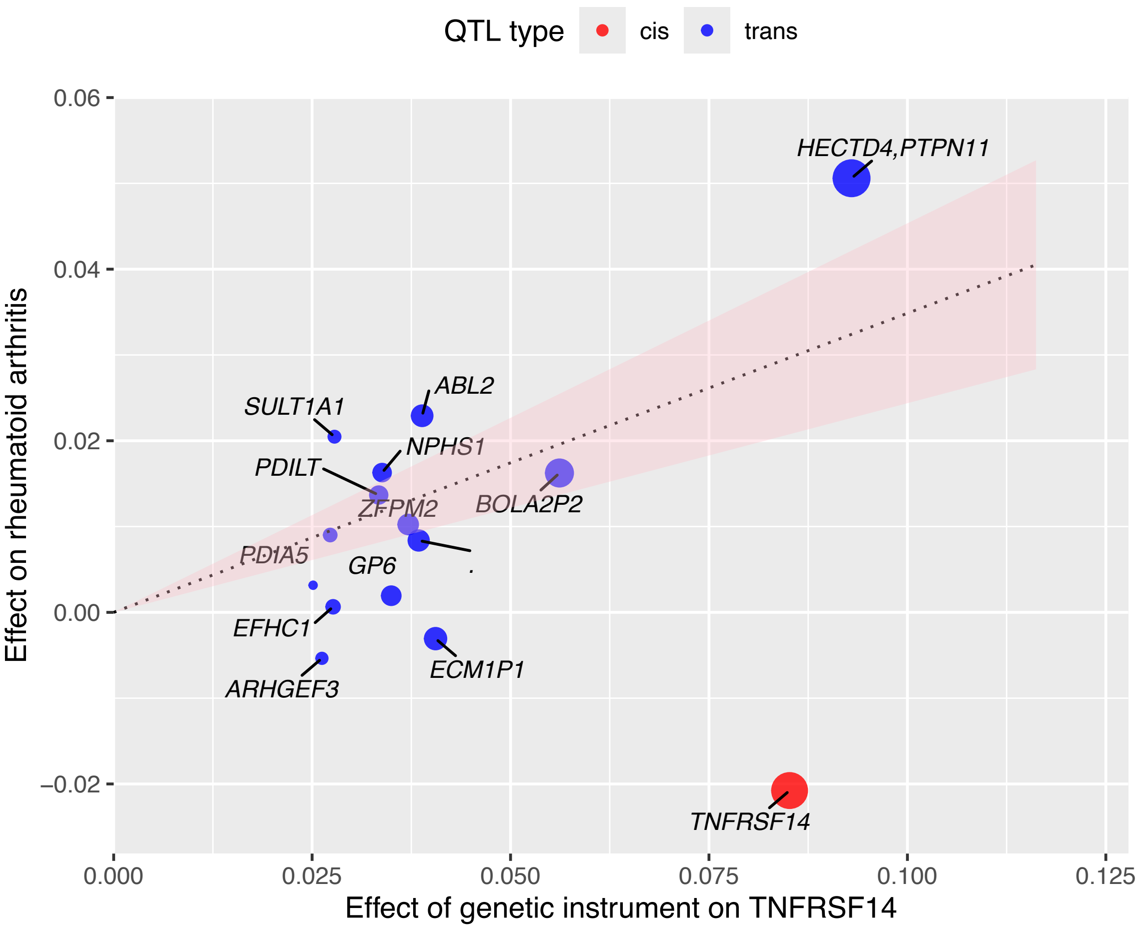

Fig S4. Mendelian randomization analysis of *TNFRSF14*. All *trans*-pQTLs with at least one SNP association with the protein at $p < 10^{-6}$ are included in the analysis, but the *cis*-pQTL (red circle) is excluded. The size of each point is inversely proportional to the standard error of the ratio estimator. The slope of the line is the maximum likelihood estimate of the causal effect parameter. The shaded ribbon is one standard error above and below the maximum likelihood estimate.

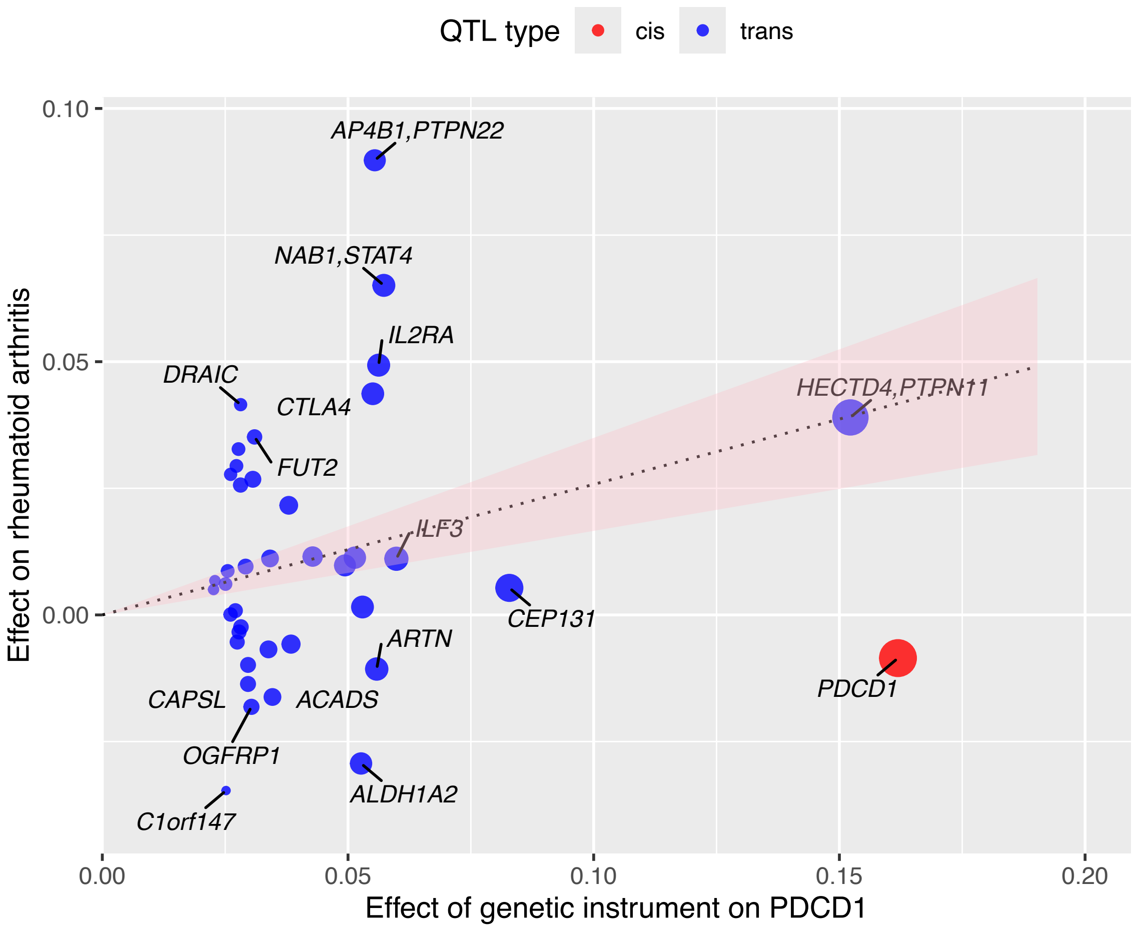

Fig S5. Mendelian randomization analysis of effect of *PDCD1* on rheumatoid arthritis. All *trans*-pQTLs with at least one SNP association with the protein at $p < 10^{-6}$ are included in the analysis, but the *cis*-pQTL (red circle) is excluded. The size of each point is inversely proportional to the standard error of the ratio estimator. The slope of the line is the maximum likelihood estimate of the causal effect parameter. The shaded ribbon is one standard error above and below the maximum likelihood estimate.

Supplementary Tables

Table S1. *Trans*-eQTLs for genes identified as putative core genes

Trans-eQTL clump	Chrom	Clump start position (Mb)	Clump end position (Mb)	Target genes	Genes in or near *trans*-eQTL clump
1	1	113.63	113.83	*CD5, CTLA4, IL10RA, SLAMF1, STAP1*	*MAGI3, PHTF1,* ***PTPN22, RSBN1***
2	2	25.27	25.27	*CD5*	*DNMT3A*
3	2	43.33	43.33	*IL10RA*	***THADA***
4	3	46.16	46.36	*SLAMF1*	***CCR1, CCR2, CCR3,*** *UQCRC2P1*
5	3	56.82	56.82	*IL10RA*	*ARHGEF3*
6	4	105.14	105.14	*CD5*	.
7	5	35.84	35.94	*CTLA4, SLAMF1*	*CAPSL, IL7R*
8	5	96.91	97.01	*CTLA4*	***ERAP2, LNPEP***
9	5	148.82	148.82	*IL10RA*	.
10	5	158.82	158.83	*STAP1*	*EBF1*
11	6	0.41	0.42	*CTLA4*	***IRF4***
12	6	27.84	27.84	*FBLN7*	.
13	6	28.34	28.34	*FBLN7*	*ZSCAN31*
14	6	29.28	29.28	*FBLN7*	*LINC03003*
15	6	31.11	32.11	*FBLN7, CTLA4, STAP1, CD5*	*ABHD16A, AIF1,* ***APOM,*** *ATP6V1G2, ATP6V1G2-DDX39B, BAG6,* ***HLA-B, HLA-C, MICA,*** ...
16	6	32.55	32.67	*CD5, CTLA4, FBLN7, SLAMF1*	***HLA-DQA1, HLA-DQB1,*** *HLA-DQB1-AS1,* ***HLA-DRB1, HLA-DRB6,*** *RNU1-61P*
17	6	90.10	90.32	*SLAMF1, CTLA4*	***BACH2,*** *MIR4464*
18	6	116.89	116.94	*IL10RA*	*RFX6*
19	6	135.42	135.42	*CD5*	*AHI1*
20	7	28.12	28.16	*FBLN7*	***JAZF1***
21	8	129.60	129.61	*STAP1*	*CCDC26*
22	10	6.05	6.07	*CTLA4*	***IL2RA***
23	10	79.27	79.27	*CTLA4*	***ZMIZ1***
24	10	89.00	89.00	*CD5*	*FAS*
25	12	6.33	6.34	*CTLA4*	***TNFRSF1A***
26	12	56.00	56.00	*CD5*	.
27	12	111.27	112.43	*FBLN7*	*ACAD10, ADAM1A, ADAM1B, ALDH2, ATXN2,* ***HECTD4, PTPN11, SH2B3,*** ...
28	12	128.80	128.80	*STAP1*	*SLC15A4*
29	14	98.02	98.03	*CD5*	.

30	17	39.76	39.93	*STAP1*	***GSDMB***, ***IKZF3***, *KRT8P34*, *LRRC3C*, ***ORMDL3***, ***ZPBP2***
31	19	16.36	16.44	*SLAMF1, FBLN7*	*EPS15L1*
32	19	19.56	19.56	*IL10RA*	.
33	20	32.79	32.79	*CD5*	***DNMT3B***
34	20	46.11	46.12	*STAP1*	***CD40***

In the clumps of *trans*-eQTLs, genes previously reported as GWAS hits are shown in bold, together with up to 5 genes not reported as GWAS hits. The *trans*-eQTL clump in the HLA region is shown in this table, but excluded from the calculation of GATE scores.

Table S2. Associations of rheumatoid arthritis in UK Biobank with polygenic scores for immune cell phenotypes derived from the SardiNIA cohort

Trait	Number of QTLs	Log odds ratio	p-value
CD4+ Treg % of CD4+ T cells	14	-0.063	3×10^{-6}
CD4+ not Treg % of CD4+ T cells	14	0.063	3×10^{-6}
CD4+ absolute count	14	0.063	6×10^{-6}
CD4+ CD8+ absolute count	15	0.057	3×10^{-5}
HLA DR+ % of NK cells	14	-0.054	7×10^{-5}
CD45RA+ CD28- CD8+ % of CD8+ cells	18	-0.052	2×10^{-4}
CD4+/CD8bright	10	-0.045	0.001

All associations with $p < 0.001$ are shown. Associations were adjusted for age, sex and the first 20 principal components of the genotype matrix.

Table S3. *Trans*-pQTLs for genes identified as putative core genes

Trans-pQTL clump	Chrom	Clump start position (Mb)	Clump end position (Mb)	Target genes	Genes in or near *trans*-pQTL clump
1	1	1.21	1.37	*PDCD1*	*ACAP3, B3GALT6, C1QTNF12, CPTP, DVL1,* ***SDF4****, ...*
2	1	43.33	44.12	*PDCD1*	*ARTN, ATP6V0B, B4GALT2, CCDC24, CDC20, ...*
3	1	109.92	109.96	*LAIR1*	*CSF1*
4	1	113.53	114.14	*PDCD1, TIGIT, CXCL9, CXCL10*	***AP4B1****, AP4B1-AS1, BCL2L15, DCLRE1B, HIPK1, HIPK1-AS1,* ***PTPN22****,* ***RSBN1****, ...*
5	1	150.58	150.72	*LILRA4*	*ENSA, GOLPH3L, HORMAD1, MCL1, RNU6-1042P, ...*
6	1	156.77	156.84	*PDCD1*	*HDGF, NTRK1, PRCC, SH2D2A*
7	1	159.17	159.47	*LILRA4*	*ACKR1, AIM2, CADM3, CADM3-AS1, FCER1A, ...*
8	1	161.12	161.22	*LILRA4*	*ADAMTS4, B4GALT3, DEDD, FCER1G, NDUFS2, ...*
9	1	161.52	161.56	*IDO1*	*FCGR3A, HSPA6, RPS23P10*
10	1	172.69	172.86	*TP53BP1*	*SLC25A38P1*
11	1	178.99	179.79	*TNFRSF14, PDCD1*	*ABL2, AXDND1, COX5BP8, EIF4A1P11, FAM163A, ...*
12	1	198.30	198.46	*PDCD1*	*NEK7*
13	1	199.01	199.04	*IDO1*	*LINC01221, LINC01222*
14	1	206.50	206.51	*PDCD1*	*C1orf147, IKBKE, RASSF5*
15	2	27.33	27.53	*CXCL9, CXCL10*	*EIF2B4, FNDC4, FTH1P3,* ***GCKR****, GTF3C2, GTF3C2-AS1, ...*
16	2	38.03	38.12	*TP53BP1*	*CYP1B1, CYP1B1-AS1, RMDN2, RMDN2-AS1*
17	2	38.69	38.71	*TP53BP1*	*GALM*
18	2	62.26	62.38	*PDCD1, TIGIT*	*RN7SL51P*
19	2	65.33	65.41	*LILRA4*	***SPRED2***
20	2	102.30	102.48	*CXCL10, CXCL9*	***IL18R1****, IL18RAP, IL1RL1, MIR4772, SDR42E1P5, SLC9A4*
21	2	110.83	111.27	*TP53BP1, LILRA4*	***ACOXL****, ACOXL-AS1, BCL2L11, MIR4435-2HG, RPL5P9*
22	2	162.10	162.27	*TNFRSF14, CXCL10*	.

23	2	190.39	191.11	*CXCL9, PDCD1, CXCL10*	*GLS, MFSD6,* ***NAB1****, NEMP2, NEMP2-DT, RAB1AP1,* ***STAT4****, ...*
24	2	203.83	203.94	*PDCD1, TP53BP1*	***CTLA4***
25	2	206.52	206.52	*TIGIT*	*ADAM23*
26	3	17.47	17.62	*LAIR1*	*TBC1D5*
27	3	45.87	47.54	*CXCL10, CXCL9, TNFRSF14*	***CCR1****,* ***CCR2****,* ***CCR3****, CCR5, CCR5AS, CCR9, CCRL2, CXCR6, ...*
28	3	56.88	56.92	*TNFRSF14, PDCD1*	*ARHGEF3*
29	3	58.20	58.20	*CXCL9*	***DNASE1L3***
30	3	101.21	101.59	*IDO1*	*FAM136CP, FAM172BP, IMPG2, PCNP, SENP7, ...*
31	3	121.79	121.98	*TIGIT*	*EAF2, IQCB1, SLC15A2*
32	3	123.11	123.14	*TNFRSF14*	*PDIA5*
33	3	159.91	159.95	*CXCL9*	*IL12A-AS1*
34	3	170.00	170.20	*PDCD1*	*GPR160, KMT5AP3, PHC3, RNU4-38P, RNU6-315P*
35	3	187.02	187.02	*PDCD1*	*ST6GAL1*
36	4	76.26	76.49	*LAIR1*	*CCDC158, FAM47E, FAM47E-STBD1, RNU6-1000P, SHROOM3, ...*
37	4	102.53	102.57	*CXCL9*	***NFKB1***
38	4	104.55	105.12	*LILRA4*	*CXXC4-AS1, RNU6-351P, RPL6P14*
39	4	121.25	121.26	*CXCL10*	.
40	4	122.07	122.64	*TP53BP1*	*ADAD1, IL2,* ***IL21****, IL21-AS1,* ***KIAA1109****, RN7SL335P*
41	4	184.24	184.32	*IDO1*	*MYL12BP2, RN7SL28P, RPL6P16*
42	5	35.80	35.93	*PDCD1*	*CAPSL, IL7R, SPEF2*
43	5	132.15	132.60	*CXCL10*	***IL5****, IRF1, IRF1-AS1, LINC02863, MIR3936, MIR3936HG, ...*
44	5	138.16	138.29	*PDCD1*	*BRD8, CDC23, CDC25C, GFRA3, KIF20A, ...*
45	5	139.46	139.47	*CXCL10*	*SMIM33*
46	5	159.34	159.40	*CXCL9*	.
47	6	0.40	0.62	*TP53BP1*	*EXOC2,* ***IRF4***
48	6	25.41	33.55	*TP53BP1, CXCL9, PDCD1, TNFRSF14, LAIR1, CXCL10, IDO1, TIGIT, LILRA4*	*ABCF1, ABHD16A, ABT1, AGER, AGPAT1,* ***APOM****,* ***HLA-B****,* ***HLA-C****,* ***HLA-DQA1****,* ***HLA-DQA2****,* ***HLA-DQB1****,* ***HLA-DRB1****,* ***HLA-DRB5****,* ***HLA-DRB6****,* ***MICA****, ...*
49	6	44.09	44.14	*LAIR1*	*MRPL14, TMEM63B*
50	6	51.28	51.30	*PDCD1*	.

51	6	52.38	52.51	*TNFRSF14*	*EFHC1, PAQR8, TRAM2*
52	6	90.27	90.30	*PDCD1*	***BACH2***
53	6	96.06	96.12	*PNLIPRP2*	*FUT9*
54	6	115.98	116.76	*IDO1*	*CALHM4, CALHM5, CALHM6, CALHM6-AS1, CBX3P9, ...*
55	6	137.62	137.69	*TP53BP1*	*LINC03004*
56	6	159.05	159.09	*PDCD1*	*TAGAP-AS1*
57	6	159.95	161.25	*CXCL9*	*AGPAT4, AIRN, CHP1P2, IGF2R, LPA, ...*
58	7	2.97	2.98	*TP53BP1*	*CARD11*
59	7	6.46	6.51	*LILRA4*	*DAGLB, GRID2IP, KDELR2*
60	7	16.89	17.48	*TP53BP1*	*AHR, BRWD1P3, LINC02888, LINC02889, Metazoa_SRP, ...*
61	7	17.72	17.98	*LAIR1*	*MRM3P2, SNX13*
62	7	28.10	28.22	*CXCL9, TP53BP1*	***JAZF1***, *RNU6-979P*
63	7	49.69	50.74	*LILRA4*	*DDC, DDC-AS1, FIGNL1, GNL2P1, GRB10, ...*
64	7	77.16	77.27	*LAIR1*	*CCDC146, FGL2*
65	7	106.67	106.74	*TNFRSF14*	.
66	7	129.10	129.10	*CXCL10*	.
67	7	150.51	150.63	*IDO1*	*ALDH7A1P3, GIMAP4, GIMAP6, GIMAP7, STRADBP1, ...*
68	8	8.67	8.78	*CXCL9*	*CLDN23*
69	8	42.12	42.25	*CXCL9*	*AP3M2, IKBKB-DT, PLAT*
70	8	60.73	60.75	*IDO1*	*CHD7*
71	8	80.23	80.48	*TP53BP1*	*MIR5708, RNU6-1213P*, ***TPD52***
72	8	105.50	105.58	*TNFRSF14*	*ZFPM2, ZFPM2-AS1*
73	8	123.61	123.66	*TIGIT*	*KLHL38*
74	8	125.36	125.36	*TP53BP1*	*NSMCE2*
75	8	143.90	144.01	*IDO1*	*GRINA, MIR661, PARP10, PLEC*
76	9	1.85	1.87	*PDCD1*	.
77	9	4.74	4.87	*TNFRSF14*	*ECM1P1, KLF4P1, MIR101-2, RCL1*
78	9	5.48	5.50	*PDCD1*	.
79	9	9.21	9.28	*TP53BP1*	*PTPRD*
80	9	32.43	32.58	*CXCL10*	*ACO1, DDX58, DFFBP1, NDUFB6, SMIM27, ...*
81	9	33.12	33.17	*LILRA4*	*B4GALT1, B4GALT1-AS1*
82	9	93.12	93.31	*IDO1*	*NINJ1, WNK2*
83	9	110.57	110.60	*CXCL10*	*SVEP1*
84	9	114.78	114.93	*TP53BP1*	*DELEC1*, ***TNFSF15***, ***TNFSF8***

85	9	120.75	120.96	*PDCD1*	*B3GALT9*, ***C5***, *CUTALP*, *FBXW2*, *PHF19*, *PSMD5*, ***TRAF1***
86	9	133.19	133.52	*PNLIPRP2*	*ABO*, *ADAMTS13*, *CACFD1*, *LCN1P1*, *LCN1P2*, ...
87	10	5.69	6.07	*PDCD1*	*ANKRD16*, *FBH1*, *GDI2*, *IL15RA*, ***IL2RA***, *NRBF2P5*, ...
88	10	8.41	8.45	*IDO1*	.
89	10	43.66	43.76	*CXCL9*	*ELOCP30*, *SPRING1P1*, *UQCRHP3*, *ZNF32-AS3*
90	10	69.38	69.54	*PDCD1*	*ATP5MC1P7*, *HK1*, *TACR2*, *TMEM256P1*, *TSPAN15*
91	10	80.20	80.55	*LILRA4*, *PDCD1*	*ANXA11*, *DYDC1*, *DYDC2*, *EIF5AP4*, *LINC00857*, ***TSPAN14***, ...
92	10	102.46	102.64	*PNLIPRP2*	*ACTR1A*, *MFSD13A*, *RNU6-43P*, *RPL23AP58*, *SUFU*, ...
93	10	103.74	103.80	*PNLIPRP2*	***SH3PXD2A***, *SH3PXD2A-AS1*
94	10	107.42	108.37	*PNLIPRP2*	*LINC01435*, *PTGES3P5*, *RNA5SP326*
95	10	109.04	109.85	*PNLIPRP2*	*BTF3P15*, *PHB2P1*, *RNU5B-6P*, *RNU6-839P*, *RPL21P91*, ...
96	10	123.04	123.52	*PNLIPRP2*	*ACADSB*, *BUB3*, *HMX2*, *HMX3*, *LINC02641*, ...
97	11	47.36	47.44	*LILRA4*	*MIR4487*, *PSMC3*, *RAPSN*, *SLC39A13*, *SPI1*
98	11	128.18	128.25	*IDO1*	*LINC02098*, *LINC02725*
99	12	10.40	10.49	*IDO1*	*KLRC1*, *KLRC2*, *KLRC3*, *KLRC4*, *KLRC4-KLRK1*
100	12	68.13	68.20	*CXCL9*, *CXCL10*	*IFNG*, ***IFNG-AS1***
101	12	109.83	112.81	*CXCL9*, *CXCL10*, *IDO1*, *PDCD1*, *TNFRSF14*, *LAIR1*, *LILRA4*, *TIGIT*	*ACAD10*, *ADAM1A*, *ADAM1B*, *ALDH2*, *ANAPC7*, ***HECTD4***, ***PTPN11***, ***SH2B3***, ...
102	12	114.38	114.38	*IDO1*	*TBX5*
103	12	120.70	120.94	*PDCD1*	*ACADS*, *ARF1P2*, *CLIC1P1*, *MIR4700*, *MLEC*, ...
104	13	27.90	29.00	*LILRA4*, *PDCD1*, *IDO1*	*ATP5F1EP2*, *CDX2*, *CHCHD2P8*, *CYP51A1P2*, *EEF1A1P3*, ...
105	13	72.32	72.32	*IDO1*	.
106	15	58.46	58.51	*PDCD1*	*ALDH1A2*, *LIPC*, *LIPC-AS1*
107	15	69.69	69.75	*PDCD1*	*DRAIC*
108	15	77.15	77.26	*PDCD1*	*PEAK1*
109	15	79.96	79.99	*IDO1*	*BCL2A1*

110	15	85.87	86.31	*LAIR1*	*AGBL1, AGBL1-AS1, LINC01584, RNA5SP400*
111	16	11.02	11.12	*PDCD1*	*CLEC16A, RPL7P46*
112	16	20.34	20.40	*LAIR1, TNFRSF14*	*PDILT, UMOD*
113	16	28.53	28.61	*TNFRSF14, LAIR1*	*NUPR1, SGF29,* ***SULT1A1****, SULT1A2*
114	16	71.42	71.89	*LILRA4*	*AP1G1, ATXN1L, CHST4, IST1, LINC02136, ...*
115	16	75.13	75.48	*PNLIPRP2*	*BCAR1, CFDP1, CHST6, CTRB1, CTRB2, ...*
116	16	85.65	85.66	*CXCL9*	*GSE1, RN7SL381P*
117	16	85.88	85.93	*TP53BP1*	***IRF8****, MIR6774*
118	16	85.98	85.99	*IDO1*	.
119	16	88.49	88.51	*IDO1*	*ZFPM1*
120	16	89.02	89.06	*LILRA4*	.
121	17	7.16	7.36	*PDCD1*	*ACADVL, ACAP1, ASGR1, CLDN7, CTDNEP1, ...*
122	17	8.14	8.26	*LILRA4*	*AURKB, BORCS6, CTC1, LINC00324, MIR4521, ...*
123	17	8.79	8.90	*TP53BP1*	*MFSD6L, PIK3R5, PIK3R6*
124	17	35.43	35.49	*CXCL9, CXCL10, IDO1*	*E2F3P1, SLFN12, SLFN12L, SLFN13*
125	17	47.33	47.73	*TP53BP1*	*EFCAB13, KPNB1, KPNB1-DT, MRPL45P2,* ***NPEPPS****,* ***TBKBP1****,* ***TBX21***
126	17	49.24	49.40	*PDCD1*	*FLJ40194, MIR6129, ZNF652, ZNF652-AS1*
127	17	62.48	62.65	*CXCL10*	*MRC2, TLK2*
128	17	64.04	64.08	*LILRA4*	***ERN1***
129	17	81.17	81.30	*PDCD1*	*CEP131, NDUFAF8, PVALEF, SLC38A10, TEPSIN*
130	18	12.77	12.88	*CXCL10, CXCL9*	*LINC01882,* ***PTPN2***
131	19	3.94	4.12	*PDCD1*	*DAPK3, EEF2, MAP2K2, MIR637, NMRK2, ...*
132	19	10.21	10.22	*IDO1*	*DNMT1*
133	19	10.29	10.49	*CXCL10, CXCL9*	*CDC37, FDX2, ICAM1,* ***ICAM3****, ICAM4, ICAM4-AS1,* ***KEAP1****,* ***RAVER1****,* ***TYK2****, ...*
134	19	10.53	11.08	*PDCD1*	*AP1M2, ATG4D, C19orf38, CARM1, CDKN2D,* ***ILF3****, ...*
135	19	35.85	35.86	*TNFRSF14*	*NPHS1*
136	19	44.89	44.93	*CXCL10*	*APOC1, APOC1P1, APOE, NECTIN2, TOMM40*
137	19	47.12	47.20	*TP53BP1*	*SAE1*
138	19	48.25	48.78	*PNLIPRP2, PDCD1*	*CA11, CARD8, CARD8-AS1, CYTH2, DBP,* ***FUT2****, ...*

139	19	53.79	54.28	*IDO1*	*CACNG6, CACNG7, CACNG8, CNOT3, LENG1, ...*
140	19	54.97	55.06	*TNFRSF14*	*GP6, GP6-AS1, NLRP2, RDH13*
141	20	10.18	10.18	*LAIR1*	*SNAP25, SNAP25-AS1*
142	21	44.29	44.29	*PDCD1*	***AIRE***
143	22	41.28	41.71	*TP53BP1*	*ACO2, C22orf46, CSDC2, DESI1, HMGN2P10, ...*
144	22	42.19	42.29	*PDCD1*	*OGFRP1, TCF20*

In the clumps of *trans*-pQTLs, genes previously reported as GWAS hits are shown in bold, together with up to 5 genes not reported as GWAS hits. The *trans*-pQTL clump in the HLA region is shown in this table, but excluded from the calculation of GATE scores

Table S4. Genes for which whole blood transcript levels in eQTLGen were associated with polygenic scores for rheumatoid arthritis

Gene	Polygenic score study PubMed ID	Assessed polygenic score effect	Test statistic	r^2	p-value
ARID5B	20453842	high PGS	5.47	0.0011	4×10^{-8}
ARID5B	20453842	high PGS	5.32	0.0011	1×10^{-7}
CTLA4	20453842	high PGS	4.92	0.0009	9×10^{-7}
CTLA4	20453842	high PGS	4.86	0.0008	1×10^{-6}
CTLA4	20453842	high PGS	4.73	0.0008	2×10^{-6}
CTLA4	20453842	high PGS	4.68	0.0008	3×10^{-6}
IL2RA	20453842	high PGS	5.70	0.0012	1×10^{-8}
IL2RA	20453842	high PGS	4.93	0.0009	8×10^{-7}
LRIG1	20453842	high PGS	5.59	0.0011	2×10^{-8}
LRIG1	20453842	high PGS	5.25	0.0010	1×10^{-7}
LRIG1	20453842	high PGS	4.73	0.0008	2×10^{-6}
MAF	20453842	high PGS	4.96	0.0009	7×10^{-7}
MILIP	24390342	low PGS	7.38	0.0037	2×10^{-13}
MILIP	24390342	high PGS	-7.27	0.0035	4×10^{-13}
MILIP	24390342	high PGS	-5.57	0.0021	3×10^{-8}
PDCD1	20453842	high PGS	7.02	0.0018	2×10^{-12}
PDCD1	20453842	high PGS	6.69	0.0017	2×10^{-11}
PDCD1	20453842	high PGS	4.86	0.0009	1×10^{-6}
RPL3P2	24390342	high PGS	6.02	0.0066	2×10^{-9}
RPL3P2	24390342	high PGS	4.69	0.0040	3×10^{-6}
SNORD63B	24390342	low PGS	4.85	0.0058	1×10^{-6}
ST8SIA1	20453842	high PGS	5.81	0.0012	6×10^{-9}
ST8SIA1	20453842	high PGS	5.35	0.0010	9×10^{-8}
STX1B	24390342	low PGS	7.50	0.0025	6×10^{-14}
STX1B	24390342	high PGS	-5.27	0.0012	1×10^{-7}
STX1B	24390342	high PGS	-4.81	0.0010	1×10^{-6}
TIGIT	20453842	high PGS	6.26	0.0014	4×10^{-10}
TIGIT	20453842	high PGS	5.24	0.0010	2×10^{-7}

These results are extracted from the Supplementary Data 5 file provided by Vosa et al. (2021) [9].

Table S5. Mendelian randomization analysis of effects of putative core genes in Table 2 on rheumatoid arthritis

Gene	QTL study	Number of QTLs	Estimate of causal effect parameter	p-value
LAIR1	UKB	11	0.437	4×10^{-4}
TP53BP1	UKB	20	0.389	6×10^{-4}
TNFRSF14	UKB	14	0.349	9×10^{-4}
LILRA4	deCODE	17	0.227	0.001
IDO1	UKB	22	0.267	0.002
PDCD1	UKB	39	0.258	0.005
CXCL9	UKB	20	0.312	0.02
CXCL10	UKB	19	0.402	0.03
SLAMF1	UKB	12	0.291	0.06

Tests are restricted to proteins with at least 10 *trans*-pQTLs The likelihood of the causal effect parameter is computed by marginalizing over the posterior distribution of the direct (pleiotropic) effects of the genetic instruments on the disease.

Table S6. Tests of association of proteins in Table 3 with incident rheumatoid arthritis

Gene	Non-cases	Cases	Log odds ratio	p-value
LGALS9	50431	269	0.50	3×10^{-16}
CXCL10	50674	271	0.40	4×10^{-11}
TNFRSF9	50067	269	0.37	2×10^{-10}
CD27	50608	271	0.35	1×10^{-9}
PDCD1	50779	274	0.35	2×10^{-9}
CXCL9	50674	271	0.36	5×10^{-9}
LAIR1	50426	269	0.29	7×10^{-8}
IL2RA	50102	265	0.32	1×10^{-7}
CD274	50067	269	0.29	1×10^{-6}
TNFRSF14	50507	270	0.25	4×10^{-5}
CD5	50790	274	0.18	0.002
CD48	50590	271	0.16	0.007
SLAMF1	49524	265	0.14	0.03
IL10RA	49524	265	0.12	0.04
CTLA4	43828	237	-0.11	0.08
TIGIT	43025	235	0.10	0.1
SLAMF7	50386	269	0.05	0.4
GP1BB	42371	236	-0.03	0.7
TP53BP1	42549	237	0.02	0.7
PNLIPRP2	50426	269	0.01	0.9
LILRA4	42371	236	-0.01	0.9
IDO1	42660	234	-0.01	0.9

Associations are adjusted for age, sex and continental ancestry. Individuals diagnosed with rheumatoid arthritis at or before baseline are excluded.

Table S7. Tests of association of rheumatoid arthritis with transcript levels in whole blood of genes in Tables 1 or 2, based on the Keio and Manchester studies

	Keio: 66 cases, 35 controls			Manchester: 55 cases, 10 controls		
Gene	Probe	Log odds ratio	p-value	Probe	Log odds ratio	p-value
CD5	230489	-1.65	4×10^{-5}	11000526	-1.12	0.03
CTLA4	221331.x	0.05	0.8	02001201	-1.37	0.007
CXCL10	204533	1.19	0.008	04001305	0.13	0.8
CXCL9	203915	0.37	0.2	04001304	0.31	0.4
FBLN7	229247	0.21	0.4	02000704	1.01	0.03
IDO1	210029	0.56	0.03	08000305	-0.06	0.9
IL10RA	204912	-1.37	8×10^{-5}	.	.	.
LAIR1	210644.s	-0.85	0.003	19001844	-1.85	0.008
LILRA4	210313	-0.57	0.04	19002720	-0.18	0.6
PDCD1	207634	-0.01	1	02002960	1.06	0.02
PNLIPRP2	.	.	.	10000846	0.85	0.07
SLAMF1	206181	-0.47	0.07	01003406	-1.90	0.008
STAP1	220059	0.63	0.03	04000362	-0.66	0.1
TIGIT	240070	0.20	0.4	03000588	-1.26	0.02
TNFRSF14	.	.	.	01000061	0.06	0.9
TP53BP1	213266	-1.01	0.001	15001278	-0.87	0.06

Log odds ratios are scaled as the difference in log odds associated with a covariate difference of one standard deviation The Keio study used the Affymetrix Human Genome U133 Plus 2.0. The Manchester study used the Affymetrix Human Transcriptome Array 2.0

Table S8. Validation of genes in Tables 1, 2 or 3 through perturbation in experimental model or drug effect in humans

Gene	Experiment	Perturbation	Mouse model of arthritis	Drug effect
IL10RA	[29]	Lentivector transduction	collagen-induced	.
CTLA4	[30]	Fusion protein	D1BC transgenic	[39]
CD5	[31]	antibody	collagen-induced	.
TP53BP1	[32]	Knockout of ligand	collagen-induced	.
TIGIT	[33]	Lentivector transduction	collagen-induced	.
TNFRSF14	[34]	Fusion protein	collagen-induced	.
LAIR1	[35]	Knockout, stimulatory antibody	collagen-induced	.
CXCL10	[36]	Antibody, retroviral transduction	collagen-induced	.
CXCL9	[37]	30C-terminal peptide	Antigen-induced	.
PDCD1	[38]	Antibody to ligand	collagen-induced	[40]
LGALS9	[44]	knockout	collagen-induced	.
IL2RA	[45]	T cells depleted of marker	athymic mice	.
TNFRSF9	[46]	Antibody	collagen-induced	.
CD274	[38]	Antibody	collagen-induced	.
CD27	[47]	Antibody to receptor	collagen-induced	.
